# Silencing SGO2 by Oxamic Acid Dissociates Glycolysis and BRCA1‐Mediated DNA Repair to Improve the Chemosensitivity of Lung Adenocarcinoma

**DOI:** 10.1002/EXP.20250098

**Published:** 2026-05-31

**Authors:** Xian Lin, Zhidan Hua, Chen Liu, Minxia Yang, Beilei Zhang, Xiaofeng Zhu, Xiao Chen

**Affiliations:** ^1^ Shengli Clinical Medical College of Fujian Medical University, Fujian Provincial Hospital, Fuzhou University Affiliated Provincial Hospital Fuzhou Fujian China; ^2^ Department of Pulmonary and Critical Care Medicine The Quzhou Affiliated Hospital of Wenzhou Medical University Quzhou People's Hospital Quzhou Zhejiang China; ^3^ Department of Oncology Molecular Oncology Research Institute The First Affiliated Hospital of Fujian Medical University Fuzhou Fujian China; ^4^ Department of Oncology National Regional Medical Center Binhai Campus of The First Affiliated Hospital Fujian Medical University Fuzhou Fujian China; ^5^ Fujian Key Laboratory of Precision Medicine For Cancer The First Affiliated Hospital of Fujian Medical University Fuzhou Fujian China; ^6^ Department of Intensive Care Unit The First Affiliated Hospital Fujian Medical University Fuzhou Fujian China; ^7^ Department of Intensive Care Unit National Regional Medical Center Binhai Campus of the First Affiliated Hospital Fujian Medical University Fuzhou Fujian China; ^8^ Department of Oral Maxillo‐Facial Surgery The First Affiliated Hospital Fujian Medical University Fuzhou Fujian China; ^9^ Department of Oral Maxillo‐Facial Surgery National Regional Medical Center Binhai Campus of the First Affiliated Hospital Fujian Medical University Fuzhou Fujian China

**Keywords:** histone lactylation, lung adenocarcinoma, oxamic acid, SGO2

## Abstract

Aerobic glycolysis and DNA damage repair participate in modulating LUAD chemo sensitivity, while the connection between glycolysis and DNA repair is not fully discovered. Here, integrated multi‐omics analyses recognized SGO2 as a glycolysis‐ and DNA repair‐associated gene. SGO2 was up regulated in lung adenocarcinoma (LUAD) compared to normal controls and independently predicted poor prognosis in LUAD patients in three independent cohorts. In addition, SGO2 compromised the cisplatin (CDDP) sensitivity of LUAD in vitro and in vivo. Mechanistically, SGO2 interacted with BRCA1 to restrain BRCA1 ubiquitination and degradation, thereby enhancing homologous recombination repair signaling. Interestingly, a dietary bioactive compound, oxamic acid (OA) served as a glycolysis inhibitor to attenuate lactate (LA) production, thereby impairing histone H3 lysine 18 lactylation (H3K18la) and histone H3 lysine 27 acetylation (H3K27ac)‐mediated chromatin accessibility to suppress SGO2 transcription. Furthermore, OA repressed SGO2/BRCA1‐regulated homologous recombination repair signaling to mitigate LUAD progression and was presented as a therapeutic compound with no apparent toxicity in vivo. This study demonstrated that SGO2 is a downstream effector of glycolysis and an upstream regulator of DNA damage repair. Silencing SGO2 with OA improved LUAD chemo sensitivity. Our work highlights the potential of SGO2 as a target for therapeutic intervention and OA as a food‐bioactive compound for LUAD treatment.

## Graphical abstract

SGO2 interacts with BRCA1 to inhibit BRCA1 ubiquitination and degradation, thereby promoting BRCA1‐induced DNA damage repair signaling and reducing the chemo sensitivity of LUADa. OA targets glycolysis to disrupt H3K18la‐ and H3K27ac‐mediated chromatin accessibility, repressing SGO2 transcription and subsequently alleviating SGO2‐mediated cancer progression.

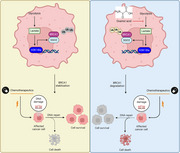

**Abbreviations**
CDDP: cisplatin
ChIP: chromatin immunoprecipitation
CHX: cycloheximide
CI: confidence interval
Co‐IP: co‐immunoprecipitation
GEO: gene Expression Omnibus
H3K18la: histone H3 lysine 18 lactylation
H3K27ac: histone H3 lysine 27 acetylation
HR: hazard ratio
IHC: immunohistochemistry
LA: lactate
LC‐MS: liquid chromatograph‐mass spectrometer
LDHA: lactate dehydrogenase A
LUAD: lung adenocarcinoma
OA: oxamic acid
PDOs: patient‐derived organoids
qPCR: quantitative real‐time PCR
ssGSEA: simple sample gene set enrichment analysis
TCGA: The Cancer Genome Atlas
TSS: transcriptional start sites
WGCNA: weighted gene co‐expression network analysis

## 1 Introduction

Lung cancer is the most commonly diagnosed malignancy with the leading mortality rate worldwide [1]. Although surgery, radiotherapy, chemotherapy, biologically targeted therapy, and other methods have been implemented to treat lung cancer, the five‐year survival rate of patients is only 23% [2]. Among them, lung adenocarcinoma (LUAD) is the most common histopathological type of lung cancer. For LUAD, surgery is the primary treatment for early‐stage disease, while advanced/metastatic cases are commonly managed with platinum‐based chemotherapy, EGFR/ALK‐targeted therapy, or anti‐PD‐1/PD‐L1 immunotherapy (first‐line for PD‐L1‐positive patients), though drug resistance remains a major clinical challenge. Currently, the high incidence and mortality rates of LUAD bring great social pressure and economic burden to the world. Exploring the molecular mechanisms contributing to LUAD chemo resistance and developing new drugs or delivery systems [3, 4] to improve the chemo sensitivity of LUAD will help ameliorate the prognosis of patients.

Aerobic glycolysis is a major metabolic feature of cancer cells. Increasing evidence supports that aerobic glycolysis promotes DNA damage repair [5]. Inhibiting the activity of lactate (LA) dehydrogenase, a glycolysis enzyme, could reduce LA production to trigger ROS production and DNA damage in cancer cells [6]. Glycolysis could promote DNA damage repair in cancer‐associated lung cancer fibroblasts [7], suggesting the influence of glycolysis on DNA repair signaling. Glycolysis also conferred chemo resistance to tumor cells through enhanced MRE11 lactylation‐mediated DNA repair [8]. Therefore, clarifying the connection between glycolysis and DNA repair might contribute to improving the chemo sensitivity of LUAD.

The human SGO2 gene encodes a protein of 1265 amino acids with two conserved domains: the N‐terminal coiled‐coil structure and the C‐terminal fundamental domain [9]. Studies have shown that SGO2 was involved in the regulation of the cell cycle and played a role in mitosis and meiosis [10]. SGO2 dysregulated the cell cycle of hepatocellular carcinoma cells by regulating MAD2‐mediated signaling to foster cancer progression [11]. Moreover, SGO2 might be a promising predictive indicator for lung cancer as it may help lung cancer cells to proliferate, migrate, and invade [12]. In prostate cancer, SGO2 stabilized RAB1A expression by inhibiting RAB1A ubiquitination to promote cell proliferation and migration [13]. However, SGO2's role in glycolysis and DNA repair in LUAD has not been elucidated.

Bioactive compounds derived from vegetables or fruits represent a broad class of dietary metabolites with potential anti‐cancer activities [14]. Dietary bioactive compounds exerted synergistic and therapeutic effects with chemotherapeutic agents through epigenetic regulation, functioning as “epidrugs” for treating various cancers [15]. Recently, our group discovered several food bioactive compounds that could potentially be used to treat cancer, including sesamolin [16] and xanthotoxol [17], and targeting glycolysis or DNA repair with such compounds is an alternative strategy. Identifying dietary bioactive compounds that modulate SGO2 or the glycolysis‐DNA repair axis might provide novel therapeutic approaches to enhance LUAD chemo sensitivity.

In the present work, our group intended to explore the link between glycolysis and DNA repair using integrated multi‐omics analyses, recognizing SGO2 as a specific gene that correlates with glycolysis and DNA repair. Thereafter, the clinical importance and function of SGO2 in LUAD were also evaluated. Besides, the effect of glycolysis on SGO2 was investigated by applying a specific glycolysis inhibitor oxamic acid (OA) to assess the possibility of utilizing SGO2 as a therapeutic target and OA as a food bioactive compound for LUAD therapy.

## 2 Results

### 2.1 Bioinformatics Analyses Indicated SGO2 as a Gene Correlated With Glycolysis and DNA Repair in LUAD

**FIGURE 1:**
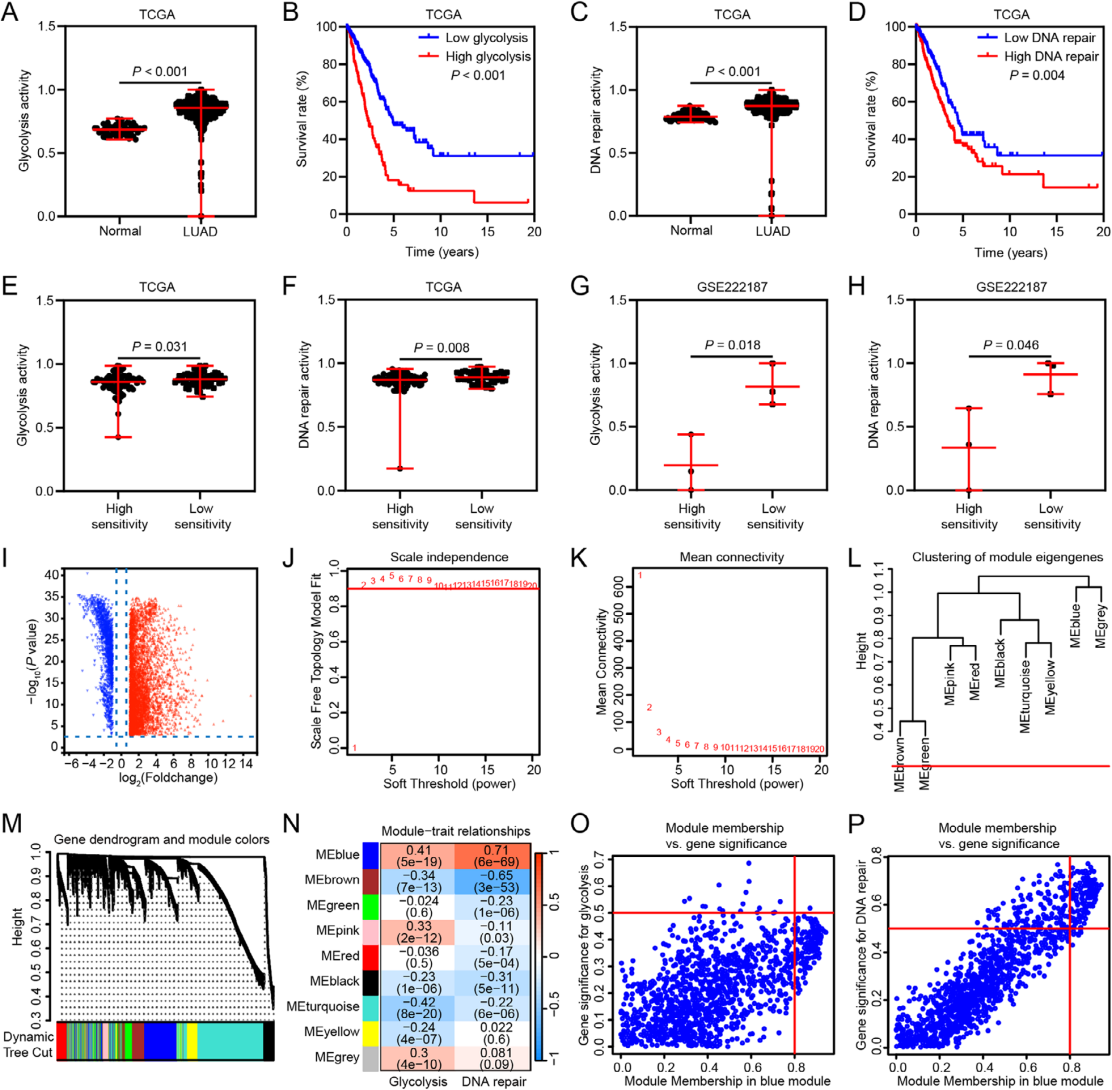
Bioinformatics analyses recognized glycolysis‐ and DNA repair‐associated genes in the TCGA LUAD dataset. (A) The differential distribution of glycolysis activity in LUAD and normal controls. (B) Survival analysis showing the association between glycolysis activity and overall survival of LUAD patients. (C) The differential distribution of DNA repair activity in LUAD and normal controls. (D) Survival analysis presenting the association between DNA repair activity and the overall survival of LUAD patients. (E) The differential distribution of glycolysis activity in LUAD with high chemosensitivity and those with low chemosensitivity. (F) The differential distribution of DNA repair activity in LUAD with high chemosensitivity and those with low chemosensitivity. (G) The differential distribution of glycolysis activity in DDP‐resistant A549 and parental A549 cells. (H) The differential distribution of DNA repair activity in DDP‐resistant A549 and parental A549 cells. (I) A volcano plot displaying the differentially expressed genes between LUAD and the normal controls. (J, K) The scale‐free topology model fit index and soft threshold were selected by portraying the scale independence and mean connectivity. (L, M) The different gene modules were shown by the gene tree (L) and delineated as the branches in the cluster dendrogram (M). (N) The connection between different gene modules and glycolysis or DNA repair was presented as the correlation coefficient and (*p* value) in the heatmap. (O, P) The scatter plots indicating the modules most significantly correlated with glycolysis and repair. LUAD: lung adenocarcinoma.

**FIGURE 2:**
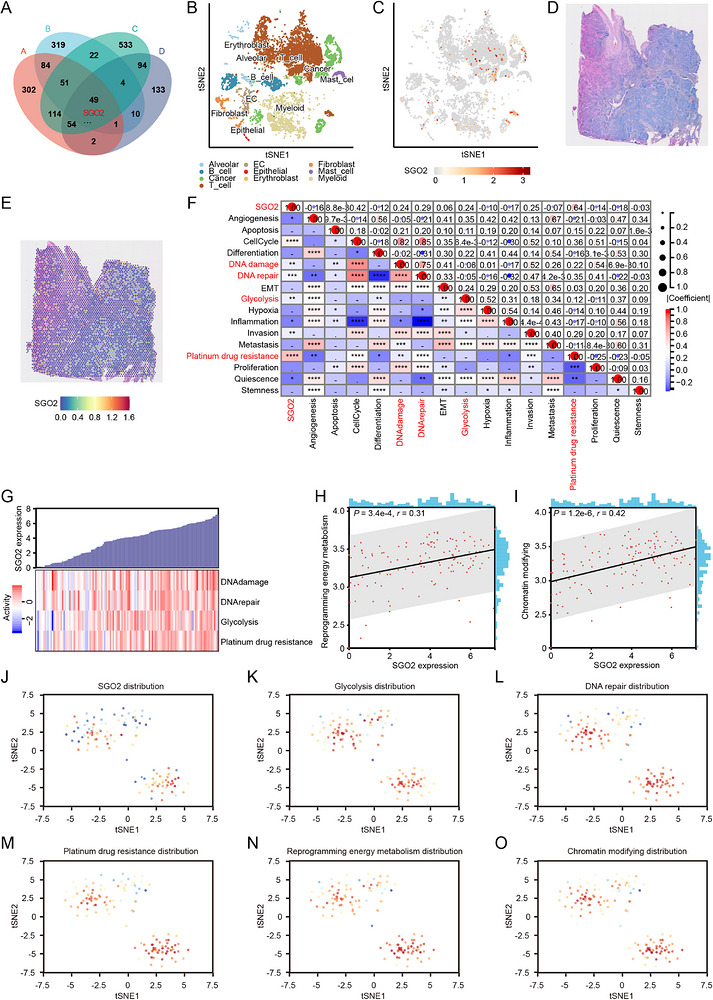
Integrated multi‐omics analyses validated SGO2 as a glycolysis‐ and DNA repair‐associated gene in LUAD. (A) A Venn diagram presenting the overlapping genes associated with glycolysis, DNA repair, and the prognosis of LUAD patients as per the TCGA and GEO databases. The glycolysis‐ and DNA repair‐related genes as per TCGA LUAD (geneset A) and GSE30219 datasets (geneset C). Genes correlated with the prognosis of LUAD patients as per TCGA LUAD (geneset B) and GSE30219 datasets (geneset D). (B, C) The t‐SNE plot based on single‐cell mRNA sequencing data showing cell types identified in the tumor microenvironment of LUAD (B) and the distribution of SGO2 expression in identified cell types from the tumor microenvironment (C). (D, E) The spatial expression of SGO2 and its distribution in LUAD. (F–I) The correlogram based on single‐cell mRNA sequencing data displaying the correlation between glycolysis, DNA repair, DNA damage, platinum drug resistance, reprogramming energy metabolism, chromatin modifying, and SGO2 expression. (J–O) The t‐SNE plot based on single‐cell mRNA sequencing data presenting the distribution of glycolysis, DNA repair, platinum drug resistance, reprogramming energy metabolism, chromatin modifying, and SGO2 expression. LUAD: lung adenocarcinoma.

To explore the connection between glycolysis and DNA repair, we adopted bioinformatics analyses to search for a specific gene bridging glycolysis and DNA repair. Glycolysis and DNA repair activity were both elevated in LUAD compared with normal controls, and both high glycolysis and DNA repair activity were positively associated with poor patient outcomes in the The Cancer Genome Atlas (TCGA) LUAD dataset (Figures 1A–D). The elevated glycolysis and DNA repair activity in LUAD and their positive connection with poor patient outcomes were validated as per the GSE30219 dataset (Figure S1A–D). Moreover, both glycolysis and DNA repair activity were elevated in LUAD samples with low chemo sensitivity compared with LUAD samples with high chemosensitivity based on the TCGA database, and both glycolysis and DNA repair activity were elevated in DDP‐resistant A549 cells compared with parental A549 cells (Figures 1E–H). Then, we combined the differential analysis with WGCNA for screening out genes correlated with glycolysis and DNA repair (Figures 1I–P and Figure S1E–L). Finally, the importance of 49 overlapping genes was evaluated by SVM‐RFE (a machine learning algorithm) (Figure S2A, B), and SGO2 ranked first among the 49 overlapping genes obtained from the intersection (Figure 2A).

Taken together, SGO2 was recognized as a glycolysis‐ and DNA repair‐related gene in LUAD.

### 2.2 Integrated Multi‐Omics Analyses Validated SGO2 as a Glycolysis‐ and DNA Repair‐Associated Gene in LUAD

SGO2 was illuminated as a glycolysis‐ and DNA repair‐associated gene (Figure 2A). Moreover, both HALLMARK and KEGG enrichment analysis confirmed the involvement of SGO2 in glycolysis and DNA repair, and SGO2 was positively correlated with glycolysis and DNA repair as per the bulk mRNA sequencing data from the TCGA LUAD and GSE30219 datasets (Figure S3A–D). Single‐cell mRNA sequencing of LUAD from a previous study [18] showed that SGO2 was mainly expressed in LUAD cells within the tumor microenvironment (Figures 2B, C). Interestingly, the spatial transcriptome of a LUAD sample from the 10x Genomics database also indicated that detectable SGO2 expression was mainly located in the tumor parenchyma (Figures 2D, E). Based on single‐cell mRNA sequencing of patient‐derived LUAD cells from another report [19], SGO2 expression was verified to be positively associated with glycolysis and DNA repair and was also found to be positively correlated with DNA damage, platinum drug resistance, reprogrammed energy metabolism, and chromatin modification (Figures 2F–O).

The findings of integrated multi‐omics analyses revealed that SGO2, expressed in LUAD cells from the tumor microenvironment, was associated with glycolysis and DNA repair.

### 2.3 SGO2 Expression Was Elevated in LUAD and Conferred Poor Patient Prognosis

Next, the clinical importance of SGO2 in LUAD was further evaluated in the TCGA LUAD dataset. In comparison to normal controls, elevated SGO2 expression was observed in LUAD (Figure S4A). Compared with early LUAD, SGO2 expression was up regulated in advanced LUAD (Figure S4B–D). Moreover, SGO2 expression was higher in patients with recurrent LUAD compared with those without recurrence (Figure S4E). Importantly, high SGO2 expression was associated with poor overall survival and recurrence‐free survival in LUAD patients (Figure S4F, G). Then, Cox analysis indicated the independent role of SGO2 expression in the prediction of overall survival (HR: 1.151, 95% CI: 1.061–1.249, *p* < 0.001) and recurrence‐free survival in LUAD patients (HR: 1.140, 95% CI: 1.045–1.243, *p* = 0.003) (Figure S4H–K). When stratified by age, gender, and TNM classification, the positive correlation remained significant between high SGO2 expression and poor overall survival and recurrence‐free survival in LUAD patients (Figure S5A–N). In addition, the relationship between tumor stage, recurrence, prognosis, and SGO2 expression was confirmed using the GSE30219 dataset (Figure S6A–F and Figure S7A–L), and SGO2 expression independently conferred poor overall survival (HR: 1.845, 95% CI: 1.297–2.625, *p* < 0.001) and recurrence‐free survival in LUAD patients (HR: 2.092, 95% CI: 1.307–3.348, *p* = 0.002) (Figure S6G–J).

**FIGURE 3:**
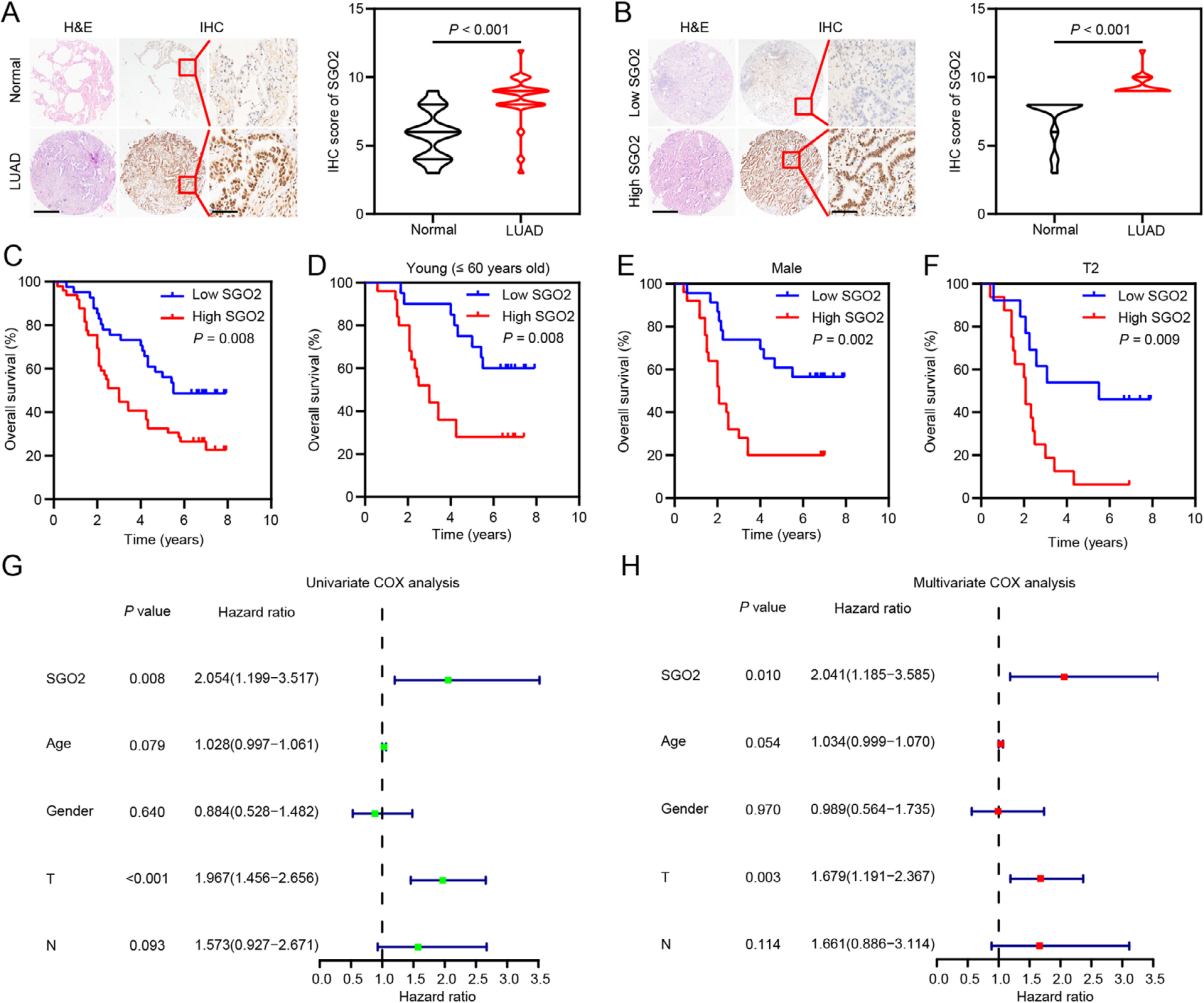
SGO2 expression measured by IHC was elevated in LUAD and predicted poor patient prognosis. (A) Representative images of LUAD samples and normal controls displaying the differential SGO2 expression. Scale bar (left): 100 µm; scale bar (right): 10 µm. (B) Representative images of LUAD samples indicating differential SGO2 expression. Scale bar (left): 100 µm; scale bar (right): 10 µm. (C) Survival analysis disclosing the correlation between patients’ overall survival and SGO2 expression in LUAD. (D–F) Subgroup analyses revealing the association between SGO2 expression and the overall survival of lung adenocarcinoma patients stratified by age (D), gender (E), and T classification (F). (G, H) Univariate and multivariate COX analysis revealing the relationship between SGO2 expression, clinicopathological features, and the overall survival of LUAD patients. IHC: immunohistochemistry; LUAD: lung adenocarcinoma.

Subsequently, an Immunohistochemistry (IHC) assay of another cohort from our lab showed that SGO2 expression was up regulated in LUAD compared to adjacent normal controls (Figure 3A and Table S1). In LUAD samples, high SGO2 expression detected by IHC predicted poor overall patient survival (Figures 3B, C). Furthermore, the association between tumor stage, prognosis, and SGO2 expression was validated in our cohort (Figures 3D–F and Table S2), and SGO2 expression independently conferred poor overall survival (HR: 2.041, 95% CI: 1.185–3.585, *p* = 0.010) (Figures 3G, H).

Collectively, the results specified SGO2 as a diagnostic and prognostic predictor in LUAD.

### 2.4 Silencing SGO2 Improved the Cisplatin Sensitivity in LUAD

**FIGURE 4:**
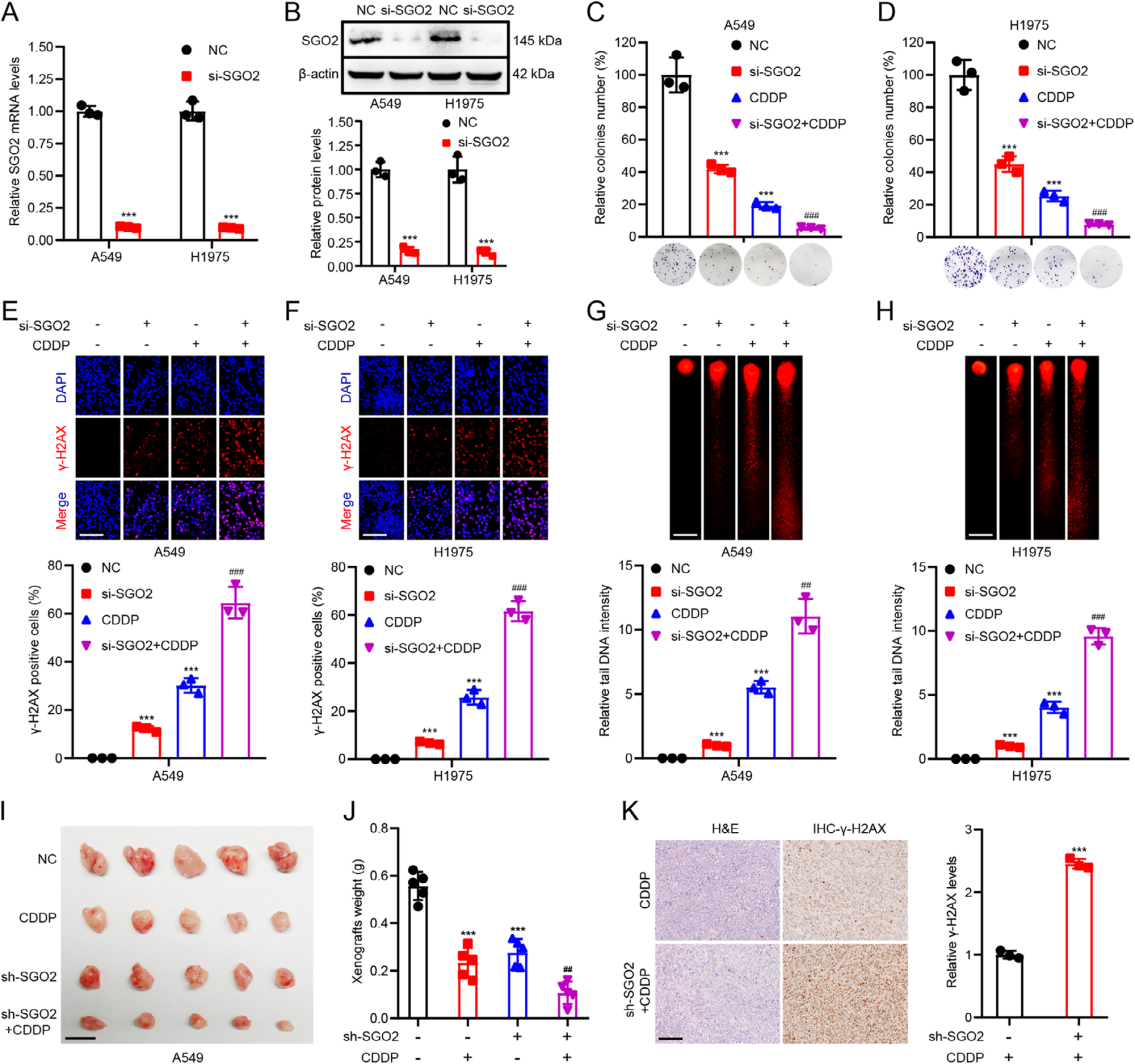
SGO2 depletion improved the chemosensitivity in lung adenocarcinoma. (A, B) qPCR and western blot analysis were adopted to measure SGO2 levels in SGO2‐depleted A549 and H1975 cells, and thecontrols. *n* = 3. (C–H) Colony formation (C, D), immunofluorescence (E, F), and comet assays (G, H) were performed to determine the chemosensitivity of SGO2‐depleted A549 and H1975 cells, and controls. Cells were treated with CDDP for 48 h and subjected to further analysis. Scale bars: 20 µm. *n* = 3. (I, J) In vivo animal study displaying the impact of SGO2 depletion and CDDP on the size and weight of tumor xenografts. Scale bar: 10 mm. *n* = 5. (K) Immunohistochemistry was applied to assess the impact of SGO2 on γ‐H2AX expression in tumor xenografts. Scale bar: 20 µm. *n* = 3. *** *p* < 0.001 vs. the control group. ^##^ *p* < 0.01, ^###^ *p* < 0.001 vs. the CDDP group. CDDP: cisplatin.

Subsequently, the function of SGO2 revealed by integrated multi‐omics analyses was experimentally analyzed in LUAD. SGO2 expression was successfully depleted using siRNA in A549 and H1975 cells (Figures 4A, B). SGO2 depletion alleviated the proliferation and improved the cisplatin (CDDP) sensitivity of LUAD cells (Figures 4C, D and Figure S8A, B). Furthermore, SGO2 knockdown increased the percentage of γ‐H2AX‐positive cells in CDDP‐treated LUAD cells (Figures 4E, F and Figure S8C, D). In addition, comet assays displayed the stimulating effect of SGO2 depletion on DNA damage of CDDP‐treated LUAD cells (Figures 4G, H). Moreover, SGO2 depletion attenuated homologous recombination repair in LUAD cells (Figure S8E). The in vivo study further revealed that silencing SGO2 could augment the CDDP sensitivity and DNA damage of LUAD xenografts (Figures 4I–K). However, silencing SGO2 did not affect the LA levels produced by LUAD cells (Figure S8F and Figure S9). Additionally, SGO2 overexpression could reverse the effects of SGO2 depletion on CDDP sensitivity and DNA repair (Figure S10A–C).

These findings collectively indicated that SGO2 impaired the CDDP sensitivity of LUAD through modulating DNA damage repair in vivo and in vitro.

### 2.5 SGO2 Interacted With BRCA1 to Restrain BRCA1 Ubiquitination and Degradation

**FIGURE 5:**
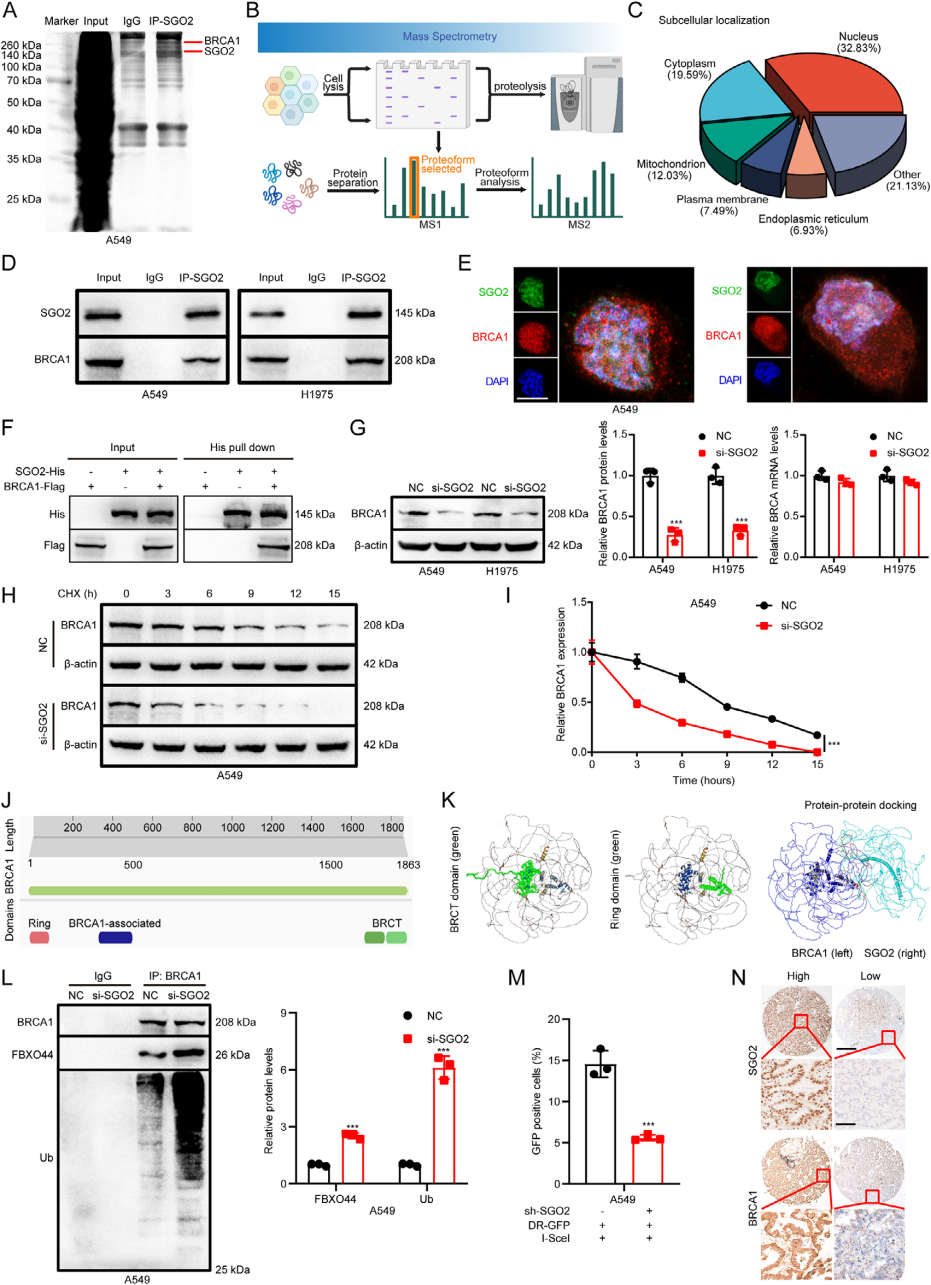
SGO2 interacted with BRCA1 to repress BRCA1 ubiquitination and degradation. (**A**) The SGO2‐interacting proteins were identified by Co‐IP combined with silver staining and mass spectrometry. (B) Method for identifying proteins using mass spectrometry. (C) The distribution of identified proteins regarding subcellular localization. (D) Co‐IP assays were applied to reveal the interplay between SGO2 and BRCA1 in A549 and H1975 cells. (E) Immunofluorescence was performed to disclose the colocalization of SGO2 and BRCA1 in the nucleus of A549 and H1975 cells. Scale bar: 10 µm. (F) His pulldown assay was perforemd to demonstrate the interaction between SGO2 and BRCA1. (G) Western blot and qPCR assays were adopted to disclose the impact of SGO2 on BRCA1 protein and mRNA expression. *n* = 3. (H, I) CHX chase assay was applied for revealing the effect of SGO2 on BRCA1 protein stability. *n* = 3. (J) The amino acid length and domains of BRCA1 protein. (K) The protein‐protein docking between BRCA1 and SGO2. (L) Co‐IP assays were performed for measuring the impact of SGO2 on BRCA1 ubiquitination in A549 cells treated with MG‐132. *n* = 3. (M) Homologous recombination reporter assays were applied to measure the effect of SGO2 on homologous recombination efficiency. *n* = 3. (N) Representative images of LUAD samples indicating the correlation between SGO2 and BRCA1 expression. Scale bar (upper): 100 µm; scale bar (lower): 10 µm. Co‐IP: Co‐immunoprecipitation; CHX: Cycloheximide.

**FIGURE 6:**
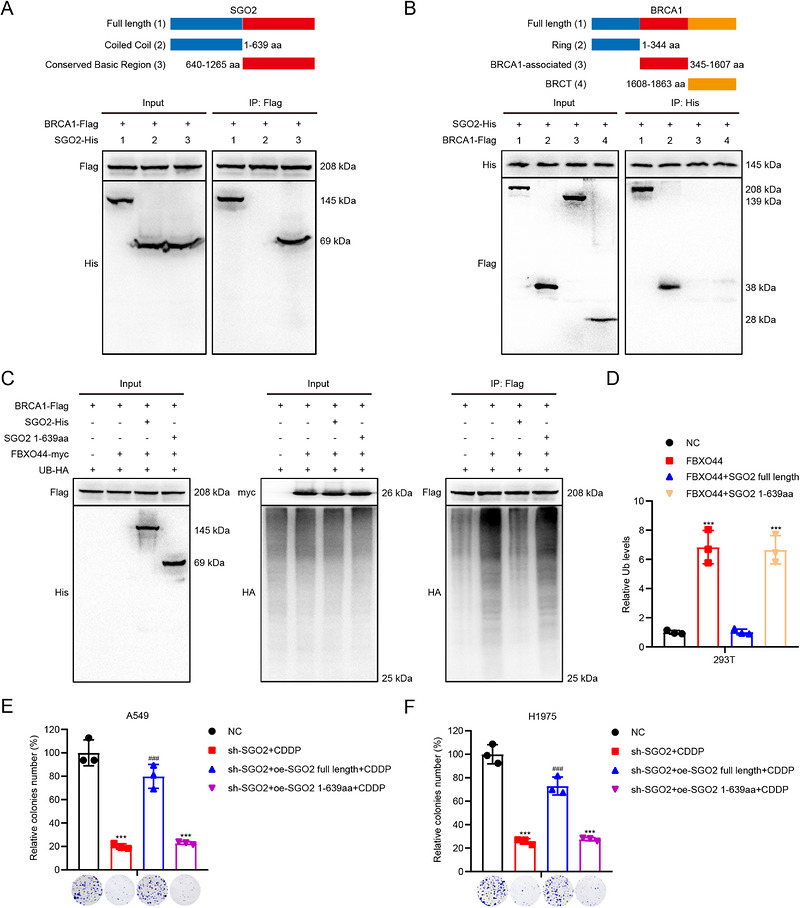
SGO2 bound to BRCA1 to attenuate the chemosensitivity in lung adenocarcinoma. (A) Co‐IP assays were applied to reveal the interplay between SGO2's conserved basic region and BRCA1 in 293T cells. Diagrammatic representation of SGO2 and its truncated forms. (B) Co‐IP assays were applied to reveal the interplay between SGO2 and BRCA1's ring domain in 293T cells. Diagrammatic representation of BRCA1 and its truncated forms. (C, D) Co‐IP assays were performed for measuring the impact of SGO2 on FBXO44‐mediated BRCA1 ubiquitination in 293T cells treated with MG‐132. *n* = 3. (E, F) Colony formation assays were performed to determine SGO2‐mediated chemosensitivity in A549 and H1975 cells, and the controls. *n* = 3. *** *p* < 0.001 vs. the control group. ^###^ *p* < 0.001 vs. the sh‐SGO2 + CDDP group. CDDP: cisplatin.

Since SGO2 contained a domain that mediated protein interactions [9], we performed co‐immunoprecipitation (Co‐IP) to enrich SGO2‐interacting proteins for mass spectrometry, and BRCA1 (an important protein for DNA damage repair) was identified as a potential SGO2‐interacting protein (Figures 5A–C). Moreover, the aforementioned analyses suggested SGO2 as a DNA repair‐associated gene, and Co‐IP further confirmed the interplay between SGO2 and BRCA1 in LUAD cells (Figure 5D). In addition, SGO2 was colocalized with BRCA1 in the nucleus of LUAD cells (Figure 5E). Moreover, His pull‐down assays confirmed the interaction SGO2 and BRCA1 (Figure 5F). Interestingly, SGO2 knockdown could impair BRCA1 protein levels but didn't affect BRCA1 mRNA expression (Figure 5G). BRCA1 ubiquitination affected its stability and degradation [20]. Then, cycloheximide (CHX) chase assays showed that silencing SGO2 decreased the half‐time of BRCA1 protein (Figures 5H, I). Protein‐protein docking showed that SGO2 could interact with the Ring domain of BRCA1 protein (binding free energy = −40 kcal·mol^−^^1^), but not with the BRCT domain (Figures 5J, K). The RING domain‐containing region of BRCA1 protein mediated BRCA1 ubiquitination and degradation by the E3 ubiquitin ligase FBXO44 [21, 22]. Furthermore, Co‐IP assays indicated that SGO2 knockdown could facilitate the interaction between BRCA1 and FBXO44 as well as the generation of BRCA1‐ubiquitin complex (Figure 5L). Moreover, silencing SGO2 impaired homologous recombination repair in LUAD cells (Figure 5M). SGO2 expression was positively correlated with BRCA1 expression as detected by IHC in LUAD samples (Figure 5N and Table S3). To further identify the binding domains, His‐tagged truncations of SGO2 and Flag‐tagged truncations of BRCA1 were constructed. Co‐IP assays revealed that BRCA1 interacted with the conserved basic region of SGO2 (Figure 6A), and SGO2 interacted with the RING domain of BRCA1 (Figure 6B). Moreover, SGO2 stabilized BRCA1 by inhibiting FBXO44‐mediated ubiquitination (Figures 6C, D). Overexpression of full‐length SGO2 attenuated the CDDP sensitivity of LUAD cells, whereas overexpression of SGO2 (amino acids 1–639) did not (Figures 6E, F).

These results collectively demonstrated that SGO2 interacted with BRCA1 to attenuate FBXO44‐mediated BRCA1 ubiquitination and degradation, thereby activating BRCA1‐mediated homologous recombination repair.

### 2.6 ChIP Sequencing Identified SGO2 as a Downstream Effector of Glycolysis

**FIGURE 7:**
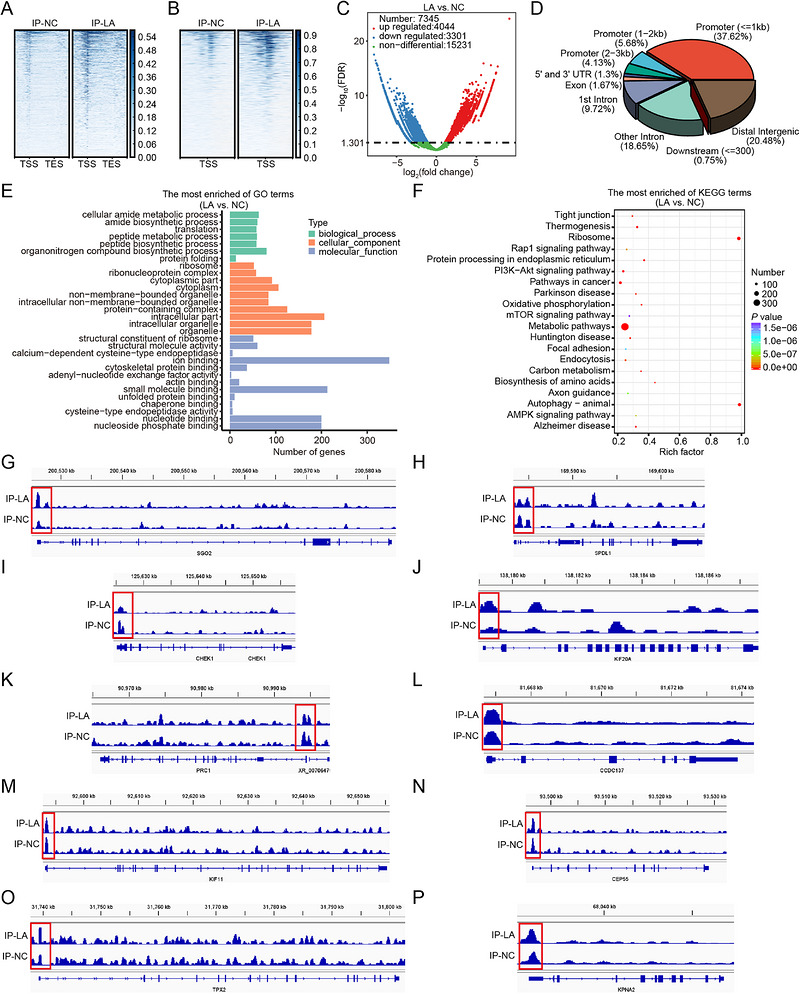
ChIP‐sequencing revealed the differential binding signal of H3K18la induced by LA treatment. (A, B) The heatmap revealing the signal distribution regarding the gene body (A) and the TSS (B). (C) A volcano plot displaying the differential peaks induced by LA treatment. (D) The distribution of differential peaks in gene functional regions. (E) Go enrichment analysis displaying the most enriched processes of the differential peak‐associated genes. (F) KEGG enrichment analysis displaying the most enriched pathways of the differential peak‐associated genes. (G–P) An integrative genomic viewer was used to visualize H3K18la binding sites within the promoter region of SGO2 (G), SPDL1 (H), CHEK1 (I), KIF20A (J), PRC1 (K), CCDC137 (L), KIF11 (M), CEP55 (N), TPX2 (O), and KPNA2 (P). LA: lactate; TSS: transcriptional start sites.

SGO2 was correlated with glycolysis and DNA repair, but we detected no effect of SGO2 on glycolysis in LUAD (Figure S9). After excluding the regulatory role of SGO2 in glycolysis, we then measured whether SGO2 could function as the downstream effector of glycolysis. Since glycolysis‐mediated histone H3 lysine 18 lactylation (H3K18la) was ubiquitously present in cancer cells and mediated epigenetic regulation of LA (LA, a product of glycolysis) metabolism [23], we conducted chromatin immunoprecipitation (ChIP) sequencing to measure the binding of H3K18la to cis‐acting elements in LUAD cells. Referring to the gene body, the differential binding signal of H3K18la induced by LA treatment was mainly located around the transcriptional start sites (TSS) (Figure 7A), especially in the 2000 bp region upstream and downstream of the TSS (Figure 7B). Moreover, LA could elevate the binding signal of H3K18la to cis‐acting elements (Figures 7A, B), and the differential peaks were mainly located within the promoter region (Figures 7C, D). Go and KEGG enrichment analysis revealed “ion binding” and “metabolic pathways” as the most enriched processes of the differential peak‐associated genes, respectively (Figures 7E, F). More importantly, SGO2 was the most significant differential gene among the top 10 of the 49 overlapping genes associated with glycolysis and DNA repair (Figure 2A and Figure 7G–P). Our ChIP sequencing data suggested that glycolysis might function as an upstream signaling of SGO2.

### 2.7 Glycolysis‐Produced Lactate Enhanced SGO2 Expression Through Facilitating H3K18la‐ and H3K27ac‐Mediated Chromatin Accessibility

**FIGURE 8:**
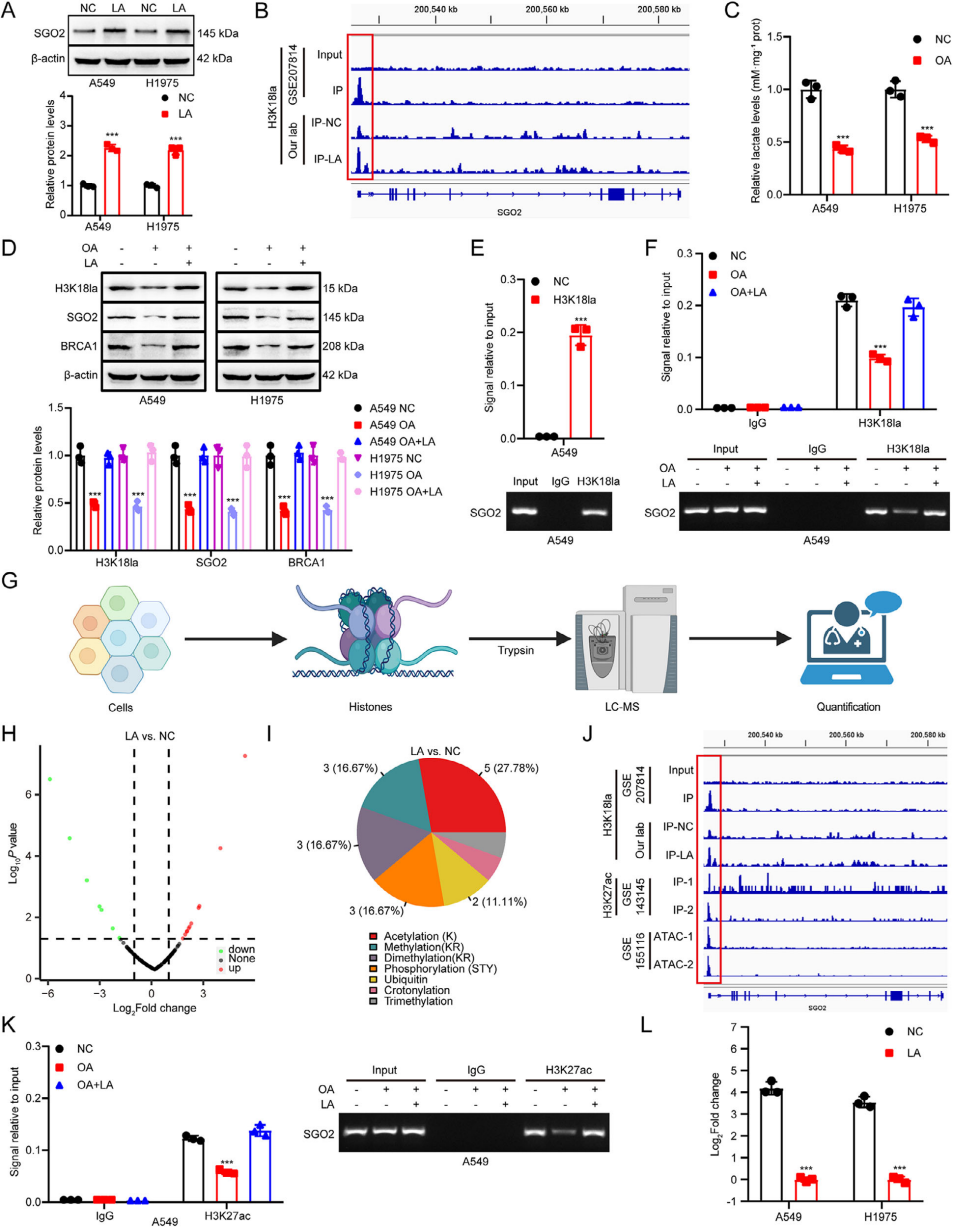
Glycolysis‐produced lactate promoted SGO2 expression through facilitating H3K18la‐ and H3K27ac‐mediated chromatin accessibility. (A) Western blot assays were adopted for evaluating the impact of LA on SGO2 expression. *n* = 3. (B) An integrative genomic viewer was used to visualize a H3K18la binding site within the promoter region of SGO2 based on ChIP‐sequencing data in the GSE207814 dataset and in our lab. (C) Lactate levels were measured in OA‐treated A549 and H1975 cells, and the controls. *n* = 3. (D) Western blot assays were applied to disclose the impact of OA and LA on the expression of H3K18la, SGO2, and BRCA1. *n* = 3. (E) ChIP assays were used for measuring the binding of H3K18la to the SGO2 promoter. *n* = 3. (F) ChIP assays were conducted to visualize the impact of OA and LA on H3K18la‐mediated SGO2 transcription. *n* = 3. (G) Experimental flowchart of histone modification identification by LC‐MS analysis. (H) A volcano plot showing the differential histone modification between LA‐treated and control LUAD cells. (I) The distribution of differential histone modification types. (J) An integrative genomic viewer was applied to visualize an overlapping binding site within the promoter region of SGO2 based on ChIP sequencing using H3K18la and H3K27ac antibodies as well as ATAC sequencing. (K) ChIP assays were conducted for displaying the impact of OA and LA on H3K27ac‐mediated SGO2 transcription. *n* = 3. (L) Chromatin accessibility assays indicating the impact of LA on the chromatin state of SGO2. *n* = 3. Cells were treated with OA and LA for 48 h and subjected to further analysis. *** *p* < 0.001 vs. The control group. LA: lactate; ChIP: chromatin immunoprecipitation; OA: oxamic acid; LC–MS: liquid chromatograph‐mass spectrometer; and LUAD: lung adenocarcinoma.

Subsequently, LA was used to test the effect of glycolysis on SGO2 expression and LA indeed augmented SGO2 expression (Figure 8A). SGO2 was potentially regulated by H3K18la according to ChIP‐sequencing data in the GSE207814 dataset and in our lab (Figure 8B). In the search for a glycolysis inhibitor, we adopted OA (oxamate) for further investigation. The selection of the OA concentration was based on previous reports [24, 25]. After uptake, OA was primarily localized in the cytoplasm (Figure S11). OA could dose‐dependently suppress the viability of LUAD cells (Figure S12A, B). Furthermore, OA impaired the LA production in LUAD cells (Figure 8C) and inhibited the expression of H3K18la, SGO2, and BRCA1, and the effect could be reversed by LA treatment (Figure 8D). Moreover, ChIP assay validated that H3K18la could bind to the promoter region of SGO2 (Figure 8E). OA could disrupt the interaction between H3K18la and the SGO2 promoter region, and the impact could be rescued by LA treatment (Figure 8F). Furthermore, OA could reverse the stimulatory effect of LA dehydrogenase A (LDHA) overexpression on H3K18la and SGO2 expression (Figure S13A, B), and H3K18la expression was positively correlated with SGO2 expression as detected by IHC in LUAD samples (Figure S13C and Table S4). In addition, we measured the landscape of histone modification by liquid chromatograph‐mass spectrometer (LC‐MS) analysis (Figure 8G). Although the abundance of LA‐induced histone lactylation was too low to detect, the histone modification omics revealed that LA treatment could increase the acetylation of histone H3K27 (histone H3 lysine 27 acetylation (H3K27ac)) (Figures 8H, I, and Table S5), a well‐known mark of active chromatin. H3K18la was reported to be capable of marking active promoters and cooperating with histone H3 lysine 27 acetylation (H3K27ac) to regulate gene expression [26, 27]. Moreover, H3K27ac could modulate chromatin accessibility which serves as a hallmark of active transcription [28, 29]. Interestingly, the enriched peak at the SGO2 promoter was also detected by ChIP sequencing using H3K18la and H3K27ac antibodies as well as ATAC sequencing (Figure 8J). Furthermore, ChIP assays confirmed that H3K27ac could be bound to the same promoter region of SGO2 as H3K18la, and LA could restore the inhibitory effect of OA on the interplay between H3K27ac and the SGO2 promoter region (Figure 8K). In addition, the chromatin accessibility assay verified that LA could alter the chromatin state of SGO2 to an open form (Figure 8L).

Collectively, these results elucidated that glycolysis promoted SGO2 expression through facilitating H3K18la‐ and H3K27ac‐mediated chromatin accessibility.

### 2.8 OA Mitigated LUAD Progression by Compromising SGO2‐Mediated Signaling

**FIGURE 9:**
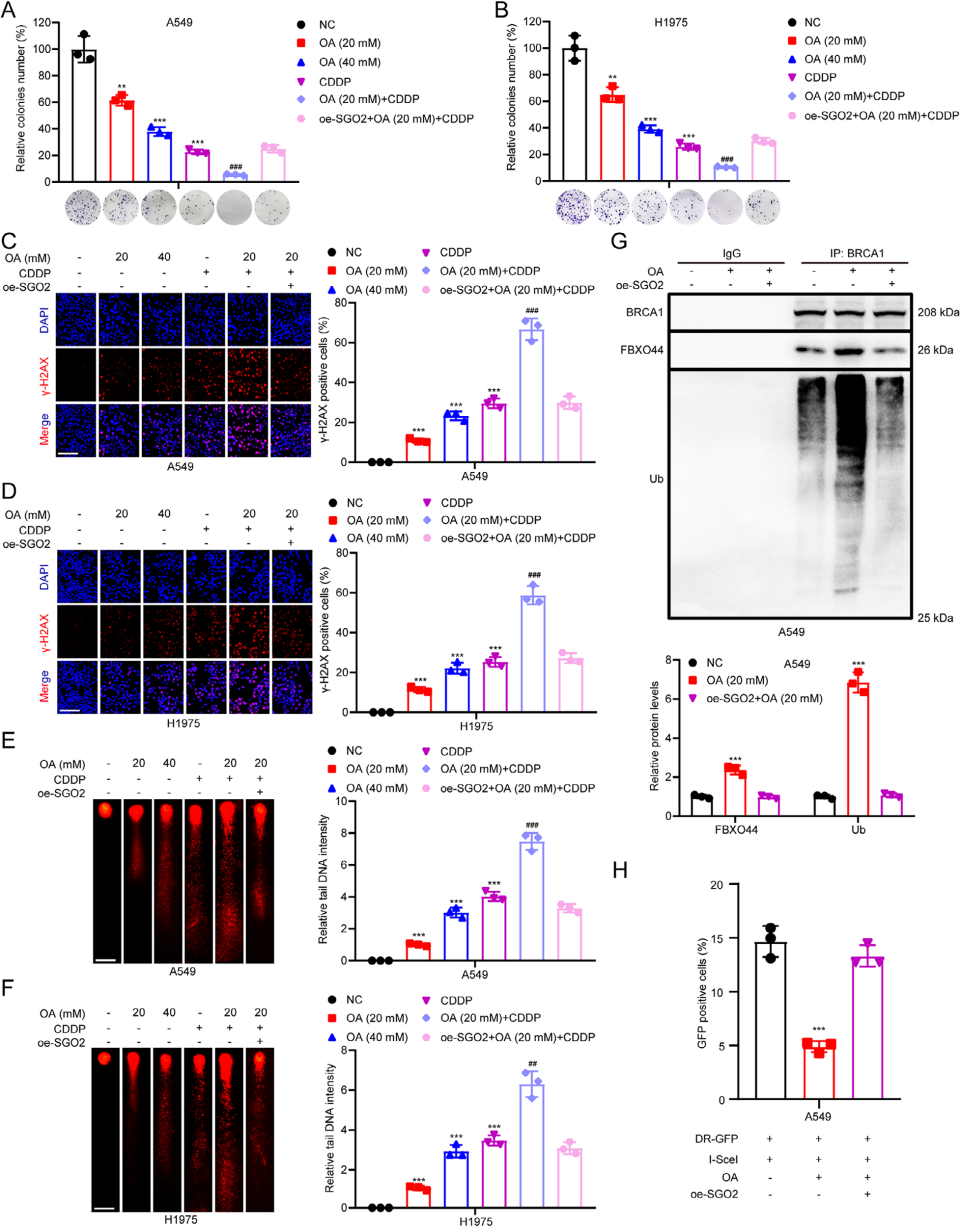
OA improved the chemosensitivity through compromising SGO2‐mediated signaling in lung adenocarcinoma. (A–F) Colony formation (A, B), immunofluorescence (C, D), and comet assays (E, F) were conducted to evaluate the effect of OA and SGO2 on the chemosensitivity of A549 and H1975 cells, and the controls. Cells were treated with CDDP for 48 h and subjected to further analysis. Scale bars: 20 µm. *n* = 3. (G) Co‐IP assays were performed for measuring the impact of OA and SGO2 on BRCA1 ubiquitination in A549 cells treated with MG‐132. *n* = 3. (H) Homologous recombination reporter assays were applied to measure the effect of OA and SGO2 on homologous recombination efficiency. *n* = 3. ** *p* < 0.01 and *** *p* < 0.001 vs. The control group. ^###^ *p* < 0.001 vs. the CDDP group. OA: oxamic acid; CDDP: cisplatin; and Co‐IP: Co‐immunoprecipitation.

**FIGURE 10:**
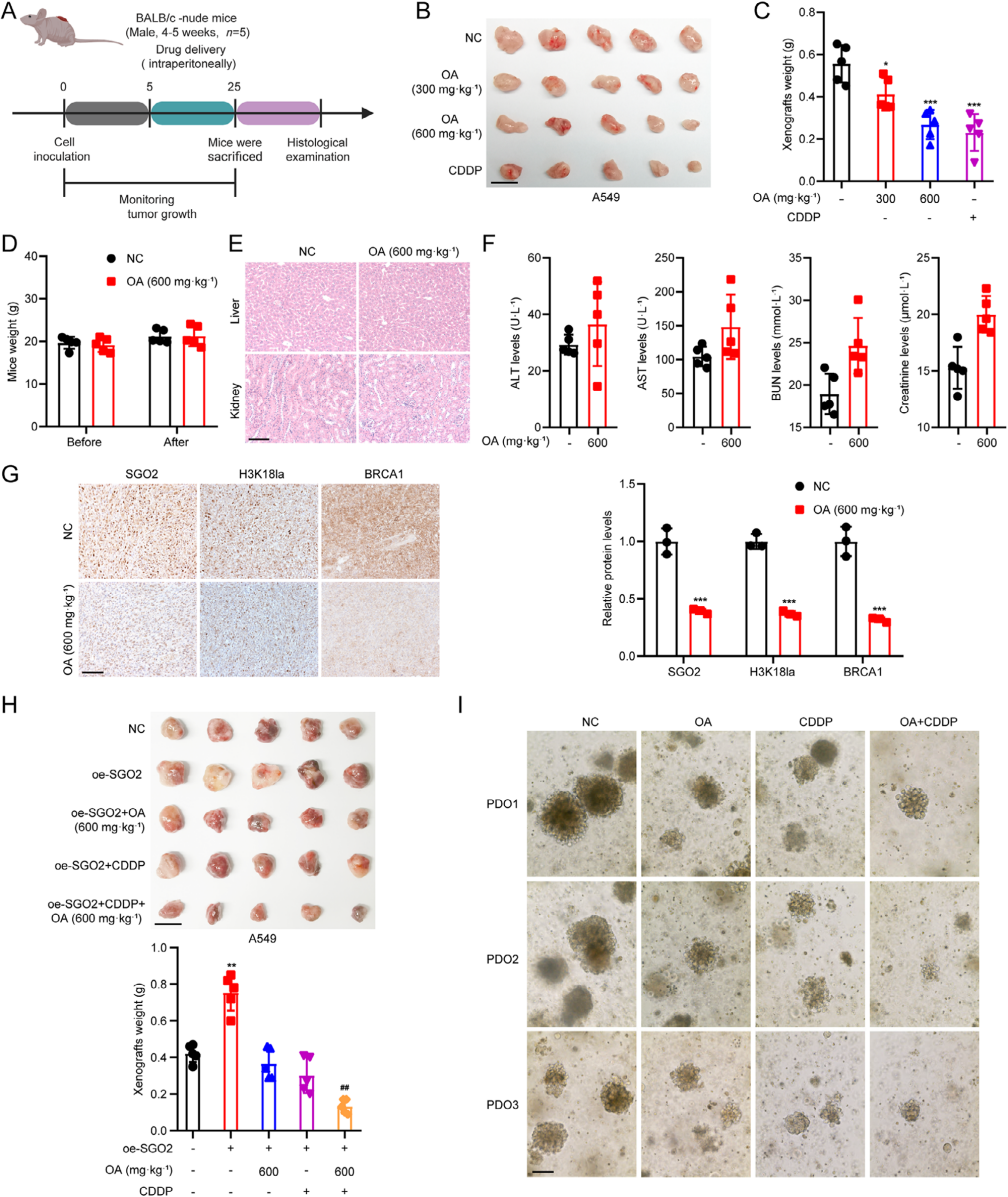
OA relieved lung adenocarcinoma progression through attenuating H3K18la/SGO2/BRCA1 signaling in vivo. (A) The designed scheme for the in vivo animal study. (B, C) In vivo animal study displaying the impact of OA on the size and weight of tumor xenografts. Scale bar: 10 mm. *n* = 5. (D) Before and after OA administration, the mice's body weight was detected to measure the in vivo toxicity. *n* = 5. (E) Histomorphological assessment of renal and hepatic architectures in OA‐treated and control groups. Scale bar: 20 µm. *n* = 5. (F) Serum biochemical testing of liver (ALT, AST) and kidney (BUN, Creatinine) in OA‐treated and control groups. *n* = 5. (G) Immunohistochemistry was conducted to assess the impact of OA on the expression of SGO2, H3K18la, and BRCA1 expression in tumor xenografts. Scale bar: 20 µm. *n* = 3. (H) In vivo animal study displaying the impact of OA and SGO2 on the chemosensitivity of tumor xenografts. Scale bar: 10 mm. *n* = 5. (I) representative bright field images displaying the impact of OA and CDDP on the growth of PDOs. Scale bar: 20 µm. *n* = 3. * *p* < 0.05 and *** *p* < 0.001 vs. The control group. OA: oxamic acid; CDDP: cisplatin; and PDOs: patient‐derived organoids.

Thereafter, we examined the role of OA in LUAD progression. OA could dose‐dependently alleviate LUAD cell proliferation, improve the CDDP sensitivity of LUAD cells, elevate the percentage of γ‐H2AX‐positive cells, and stimulate DNA damage of CDDP‐treated LUAD cells (Figures 9A–F). Moreover, OA could facilitate BRCA1 ubiquitination and impair homologous recombination repair in LUAD cells (Figures 8G, H). The inhibitory impact of OA on LUAD progression could be restored by SGO2 overexpression (Figures 9A–H). The in vivo animal study indicated that OA exhibited a suppressive role in controlling the size and weight of tumor xenografts (Figures 10A–C). By evaluating the mice's body weight and major organs, OA treatment presented no apparent toxicity in vivo (Figures 10D–F). Moreover, IHC assays of the tumor xenografts verified that OA could relieve the expression of SGO2, H3K18la, and BRCA1 (Figure 10G). Furthermore, OA enhanced the chemo sensitivity of SGO2‐overexpressing tumor xenografts (Figure 10H). In addition, OA could inhibit the growth and enhance the CDDP sensitivity of patient‐derived organoids (PDOs) (Figure 10I).

The obtained results specified that OA repressed LUAD progression by weakening SGO2/BRCA1 signaling.

## 3 Discussion

Resistance to CDDP‐based chemotherapeutics confers poor prognosis [30, 31, 32, 33]. Epigenetic modifications were capable of supporting cancer chemo resistance [34, 35]. In the present exploration, we applied integrated multi‐omics analyses to uncover a specific gene linking glycolysis and DNA repair, and elucidated SGO2 as a novel glycolysis‐ and DNA repair‐related gene. Importantly, SGO2 was independently predictive of poor prognosis in LUAD patients based on 3 independent cohorts. Furthermore, SGO2 minimized LUAD chemo sensitivity by triggering BRCA1‐mediated homologous recombination repair. In addition, the dietary supplement OA, also a glycolysis inhibitor, could restrain LA production to directly impair H3K18la and H3K27ac‐mediated chromatin accessibility, thereby repressing SGO2 transcription to inhibit LUAD progression.

In recent years, metabolic reprogramming‐regulated DNA repair in therapy resistance has attracted increasing attention [8, 36]. Based on the calculated activities, we further probed into the link between glycolysis and DNA repair by searching for a specific gene. SGO2 was screened out as a glycolysis‐ and DNA repair‐related gene, and integrated multi‐omics analyses based on bulk and single‐cell RNA mRNA sequencing as well as the spatial transcriptome of LUAD samples verified the association in LUAD cells from the tumor microenvironment. The obtained results prompted us to further investigate the role of SGO2 in LUAD cells, but not in other cell types from the tumor microenvironment. Furthermore, SGO2 was up regulated in LUAD and independently predicted poor patients’ prognosis by using 3 cohorts from our lab and the TCGA and gene expression omnibus (GEO) databases, further suggesting SGO2 as a promising indicator in the diagnosis and prognostic evaluation of LUAD.

DNA repair serves as a major determinant of cancer chemo resistance [37]. CDDP‐based chemotherapeutics are the first‐line regimen for LUAD chemotherapy [30]. By applying CDDP to induce DNA damage, we found that SGO2 could mitigate CDDP‐induced DNA damage and attenuate the chemo sensitivity of LUAD in vitro and in vivo. BRCA1 is a well‐known surveillant in maintaining genomic stability via participating in homologous recombination repair. Previous studies suggested that BRCA1‐mediated DNA damage repair conferred poor prognosis to lung cancer patients receiving platinum‐based therapy [38, 39]. Since SGO2 modulated CDDP‐induced DNA damage, we searched for a potential effector of SGO2 and determined BRCA1 as an SGO2‐interacting protein in further exploration. Moreover, SGO2 could up regulate BRCA1 expression at the post‐transcriptional level. BRCA1 ubiquitination affected its stability and degradation [20, 22, 40]. Indeed, we found that SGO2 increased the protein stability of BRCA1 through the impeding ubiquitin‐mediated BRCA1 degradation and activated BRCA1‐mediated homologous recombination repair. Another report also identified SGO2 as a stabilizer of the RAB1A protein to promote prostate cancer progression [13]. SGO2 was previously known as a protector of centromeric cohesion to modulate sister chromatid segregation [10], and loss of SGO2 could induce M‐phase arrest [41]. High SGO2 expression was correlated with poor overall survival of hepatocellular carcinoma [12]. Moreover, G2/M cell cycle arrest could affect the chemo sensitivity of LUAD cells [42]. In this work, we found that SGO2 could facilitate homologous recombination‐mediated repair to attenuate LUAD chemo sensitivity. Homologous recombination predominantly occurs in the S and G2 phases of the cell cycle [43], suggesting that SGO2 could attenuate LUAD chemo sensitivity independently of cell cycle regulation. Our present work enriched the mechanism of the regulation of chemo sensitivity by SGO2.

LA metabolism‐mediated histone lactylation which is prevalent in various cancers could promote downstream gene expression to foster drug resistance [44]. In this context, the lactylation at the H3K18 site was universally observed in cancer cells [23]. A previous report suggested that LA served as a key fuel source for TCA cycle metabolism, especially for citrate production [45]. The key aspect of citrate is to undergo mitochondrial export and subsequent cleavage by ATP‐citrate lyase to generate cytosolic acetyl‐coenzyme A (acetyl‐CoA) for histone acetylation in the nucleus. Therefore, LA could promote H3K27 acetylation via acetyl‐CoA production. Moreover, OA (a glycolysis inhibitor) could down regulate H3K27 acetylation through impairing acetyl‐CoA metabolism [46]. In the present work, we demonstrated that LA promoted SGO2 expression through H3K18la and H3K27ac‐mediated chromatin accessibility, supported by the evidence that H3K18la and H3K27ac bound to the same promoter region of SGO2 with an open form. These findings are consistent with a previous report suggesting that glycolysis enhanced cellular LA and acetyl‐CoA levels, thereby promoting H3K18la and H3K27ac [27]. Although LA could facilitate H3K18la‐ and H3K27ac‐mediated transcription as determined in our present work, we emphasized that H3K18la might play a dominant role in regulating the transcription of target genes compared to that of H3K27ac [47]. BRCA1 could bind the MRE11‐RAD50‐NBS1 complex and CtIP to form the BRCA1‐C complex, which was reported to be essential for homologous recombination‐mediated DNA double‐strand break repair [48]. Previous reports showed that glycolysis‐derived lactate facilitated the lactylation of MRE11 [8] and NBS1 [36], thus promoting homologous recombination repair and chemo resistance. In our work, we found that OA could impair the LA‐mediated H3K18la‐ and H3K27ac‐SGO2 axis to down regulate SGO2 expression, thus facilitating BRCA1 ubiquitination and degradation to improve chemo sensitivity in LUAD. Our present work elucidated the regulatory effect of LA on the BRCA1‐C complex from another direction and enriched the mechanism of LA's regulation of chemo sensitivity. In line with a previous report revealing that ubiquitination could link cancer metabolism and DNA damage repair signaling [49], we demonstrated that SGO2‐mediated BRCA1 ubiquitination might help to better understand the correlation between glycolysis and DNA damage repair.

OA (oxamate, OA), a natural product, can be obtained from Phaseolus vulgaris and Apis cerana. Phaseolus vulgaris is a traditional edible legume and a food crop with important nutritional and economic value worldwide [50]. OA served as a specific glycolysis inhibitor by targeting LDHA and exhibited anti‐cancer activities against human cancers [51, 52, 53]. Consistent with previous studies reporting that OA could improve the drug chemotherapeutic efficacy [54, 55], we found that OA could enhance the chemo sensitivity of LUAD by suppressing SGO2/BRCA1‐mediated signaling in this work. Moreover, OA could repress LUAD progression without obvious side effects, further implying that OA is a promising compound for LUAD therapy and could be utilized as a lead compound for drug development. This study has several limitations. First, it lacks validation of the SGO2/BRCA1 axis and OA's efficacy in more diverse LUAD subtypes. Second, the in vivo experiments used only xenograft models, without spontaneous tumor models to mimic clinical conditions. Third, the correlation between SGO2 expression and clinical chemotherapy response requires more prospective patient data.

In this investigation, we applied integrated multi‐omics analyses to identify SGO2 as a glycolysis and DNA repair‐related gene that independently correlated with poor patients’ survival in LUAD. Moreover, SGO2 alleviated BRCA1 ubiquitination and degradation via interaction with BRCA1, thus facilitating BRCA1‐mediated homologous recombination repair signaling to suppress LUAD chemo sensitivity. In addition, targeting glycolysis by OA could inhibit SGO2 expression through impairing H3K18la‐ and H3K27ac‐mediated chromatin accessibility, further suppressing LUAD progression. Our study indicates SGO2 as a novel target and emphasizes OA as a valuable food‐bioactive compound for the treatment of LUAD.

## 4 Methods

### 4.1 Data Acquisition and Integrated Multi‐Omics Analyses

Bulk RNA‐sequencing FPKM data and clinicopathologic characteristics in LUAD patients were extracted from TCGA database (https://portal.gdc.cancer.gov/). Data in the GSE222187, GSE30219, GSE207814, GSE143145, and GSE155116 datasets were downloaded from the GEO database (https://www.ncbinlm.nih.gov/geo/). Single‐cell mRNA sequencing data of LUAD were retrieved as indicated in previous studies [18, 19]. The spatial transcriptome of a LUAD sample was retrieved from the 10xgenomics database (https://www.10xgenomics.com/).

Data sorting was performed as previously described [56], and the obtained data were subjected to further analysis by applying the differential expression analysis, survival analysis, gene set enrichment analysis (GSEA), simple sample GSEA (ssGSEA), and weighted gene co‐expression network analysis (WGCNA). Survival analyses were conducted by plotting survival curves in low and high expression groups defined as per the best cut‐off value. As previously reported [16], the ssGSEA algorithm using the GSVA package in R was used to calculate the activity of glycolysis, DNA repair, and other functional states based on the HALLMARK and KEGG gene sets extracted from GSEA (https://www.gsea‐msigdb.org/gsea) and KEGG (https://www.genome.jp/kegg/). The WGCNA algorithm using the WGCNA package in R was applied to build a gene coexpressing network for presenting genes correlated with glycolysis and DNA repair. The machine learning algorithm SVM‐RFE was conducted using the e1071 package in R for determining the importance of genes. Single‐cell mRNA sequencing data and spatial transcriptome were analyzed using the Seurat package in R.

### 4.2 Chemical Compounds, Cell Culture, and Transfection

CDDP (CAS: 15663‐27‐1, purity: 99.63%), LA (CAS: 50‐21‐5, purity: 98.60%), and MG‐132 (CAS: 133407‐82‐6, purity: 99.99%) were purchased from TargetMol Biotechnology Corporation (Boston, USA). OA (CAS: 471‐47‐6, purity: ≥98%) was purchased from Sigma‐Aldrich (St Louis, USA). CY3‐NH_2_ (10 mg) in DMF was mixed with DMF‐dissolved OA (1.2 eq.), followed by EDC (3 eq.) and DMAP (0.5 eq.). After 6 h stirring at room temperature, the mixture was concentrated, redissolved in DCM, washed (10% citric acid, water), dried, and then filtered. The filtrate was purified by silica gel chromatography, concentrated, dispersed in ethanol, precipitated in ether, washed, and vacuum‐dried to yield CY3‐OA (Purity: ≥98%).

A549, H1975, PC‐9, and H1650 LUAD cell lines as well as the 293T cell line (STR verified) without mycoplasma contamination were purchased from Procell Company (Wuhan, China) and grown in RPMI‐1640 medium containing 10% fetal bovine serum. siRNAs were designed by the RiboBio company (Guangzhou, China). Lentivirus harboring SGO2‐targeting shRNAs, SGO2 overexpression lentivirus, DR‐GDP plasmid, and I‐SceI endonuclease plasmid were designed by Genechem company (Shanghai, China). Plasmids were purchased from Vigene Biosciences Company (Shandong, China). In line with the manufacturer's instructions, siRNAs and plasmids were introduced into cells utilizing Lipofectamine 3000, and lentivirus was transfected into cells utilizing polybrene.

### 4.3 Quantitative Real‐Time PCR (qPCR)

LUAD cells were collected to isolate total RNA. Thereafter, the isolated RNA was used for the reverse transcription to generate cDNA. Using cDNA, qPCR reagents, and specific primers (Table S5), qPCR assays were conducted on the Roche Lightcycler 480II to measure the relative mRNA levels utilizing the 2^−ΔΔCt^ method.

### 4.4 Western Blot Analysis

LUAD cells were harvested for protein extraction and quantification. After denaturation, electrophoretic separation, and transfer to a PVDF membrane, proteins were then incubated with primary antibodies (Table S6), followed by incubation with secondary antibodies. The chemiluminescence method was adopted to measure the signal reflecting protein expression, and images were recorded by Bio‐Rad ChemiDocTM XRS+ (Bio‐Rad, USA).

### 4.5 Colony‐Formation Assay

The treated LUAD cells and corresponding controls (1000 cells/well) were plated in 6‐well plates for 14 days of cultivation, and the generated colonies were fixed and subjected to staining. Images were recorded, and colony counts were quantified and analyzed.

### 4.6 Immunofluorescence

LUAD cells (5000 cells/well) were plated into 96‐well plates for treatment or left untreated. Thereafter, cells were fixed, permeabilized, and stained with primary antibodies (Table S6). For the detection of γ‐H2AX, co‐staining was then implemented with a goat anti‐rabbit IgG H and L (Alexa Fluor 594) secondary antibody and DAPI, followed by image recording with the fluorescence microscope. For the detection of the colocalization of SGO2 and BRCA1, co‐staining was then implemented with a goat anti‐rabbit IgG H and L (Alexa Fluor 488) secondary antibody, a goat anti‐mouse IgG H and L (Alexa Fluor 594) secondary antibody, and DAPI, followed by image recording with a confocal microscope.

### 4.7 Comet Assay

A comet assay kit purchased from Beyotime Corporation (Shanghai, China) was used according to the manufacturer's instructions. Normal melting point agarose was gelled on slides. The treated LUAD cells and corresponding controls were resuspended with PBS and low melting point agarose the generating the second gel covering the entire first layer of normal melting point agarose. Then, the embedded cells were treated with lysis buffer and subjected to DNA unwinding under alkaline conditions. After electrophoresis, the slides were treated with a neutral buffer, and the DNA was stained with propidium iodide solution for imaging under a fluorescence microscope. Tail DNA intensity was calculated to assess DNA damage.

### 4.8 Lactate Detection

A LA colorimetric assay kit was purchased from Elabscience Corporation (Wuhan, China) for LA detection according to the manufacturer's instructions. Briefly, cells were plated in 6‐well plates for treatment. After incubation, the supernatant was collected for measuring the concentration of LA. Cells were left for protein extraction and quantification. Relative LA levels were calculated as the concentration of LA in the supernatant/the concentration of protein in the cells.

### 4.9 CCK‐8 Assay

A CCK‐8 kit was purchased from APExBIO (Houston, USA) to detect cell viability. LUAD cells (3000 cells/well) were seeded into 96‐well plates for treatment or left untreated, and subsequently incubated with a CCK‐8 reagent for 1.5 h at 37°C, followed by the detection of absorbance values using a microplate reader.

### 4.10 Co‐Immunoprecipitation (Co‐IP)

The assay was implemented with a Pierce Co‐IP kit purchased from Thermo Scientific Corporation (Shanghai, China). Briefly, the treated cells and corresponding controls were prepared for protein extraction and quantification. Thereafter, agarose‐conjugated primary antibodies (Table S6) were reacted with 5 mg of total proteins pre‐treated with negative control agarose. After immunoprecipitation, the bound proteins were harvested for silver staining, mass spectrometry, or western blot as previously described [16]. For mass spectrometry, proteins were digested with enzyme before peptide extraction, and LC‐MS analysis was performed by Novogene Bioinformatics Technology (Beijing, China).

### 4.11 His Pull‐Down Assay

His pull‐down assays were performed as previously described [57]. Briefly, purified His‐tagged SGO2 protein was incubated overnight with cell lysates from 293T cells transfected with Flag‐tagged BRCA1. Bound proteins were eluted and analyzed via Western blot.

### 4.12 Cycloheximide (CHX) Chase Assay

A CHX chase assay was implemented for measuring protein stability as previously described [58]. The treated LUAD cells and corresponding controls were incubated with CHX (50 µg·mL^−^^1^) for the indicated time. Subsequently, cells were subjected to protein extraction and quantification, followed by the detection of the protein half‐life by western blot.

### 4.13 Protein‐protein Docking

The AlphaFold protein structures of BRCA and SGO2 were downloaded from the UniProt database. Docking was then performed with GRAMM‐X and visualized by PDBePISA. The domains of the BRCA protein were marked for displaying the interaction between BRCA and SGO2.

### 4.14 Homologous Recombination Reporter Assay

Homologous recombination repair activity was measured as previously reported [59]. Cells were co‐transfected with DR‐GDP and I‐SceI endonuclease plasmid plasmids to induce homologous recombination repair. After transfection, the percentage of GFP‐positive cells was detected by flow cytometry to reflect homologous recombination efficiency.

### 4.15 Chromatin Immunoprecipitation (ChIP)

An enzymatic chromatin IP kit purchased from CST Corporation (Boston, USA) was applied for ChIP as previously described [60]. Briefly, chromatin from LUAD cells was cross‐linked and prepared for chromatin extraction. Subsequently, the extracted chromatin was subjected to fragmentation and immunoprecipitation with anti‐H3K18la or anti‐H3K27ac‐bound magnetic beads (Table S6). An IgG antibody was used as a negative control. Then, DNA was released from the chromatin by digestion and subjected to purification. The enriched DNA was subjected to sequencing, qPCR, or PCR.

### 4.16 ChIP Sequencing

Novogene Bioinformatics Technology (Beijing, China) carried out the ChIP sequencing. Briefly, DNA quantification and qualification were conducted, followed by library preparation and quantification. After quality control, data analysis (including reads mapping to the reference genome, fragment size estimation, peak detection, peak annotation, and differential peak analysis) was performed by the bioinformatics team.

### 4.17 Histone Modification Identification by LC‐MS Analysis

The identification of histone modification was carried out by the Novogene Bioinformatics Technology (Beijing, China). LUAD cell samples were prepared for histone extraction, quantification, detection, enzymatic digestion, fraction separation, and mass spectrometry detection. Data quality and quantity were evaluated before subsequent information analysis. The raw files detected by mass spectrometry were used for protein identification based on the search results of the PEAKS database. Quantitative analysis and differential analysis were performed on the identified histone modification sites. Moreover, the expression pattern clustering analysis and volcano map analysis were conducted on the differential histone modification sites.

### 4.18 Chromatin Accessibility Assay

A chromatin accessibility assay kit purchased from Abcam Corporation (Cambridge, UK) was adopted for conducting the assay following the manufacturer's instructions. Briefly, chromatin was extracted from lysed LUAD cells and prepared for digestion in a nuclease reaction mix (or a non‐nuclease control mix for controls). Then, the proteinase K was used to release DNA from the digestion product. After the purification and elution, DNA was subjected to qPCR with specific primers (Table S5), and the ratio of nuclease‐treated to non‐nuclease control was calculated to evaluate the chromatin state.

### 4.19 In Vivo Animal Studies

The experimental protocols were authorized by the Institutional Animal Care and Use Committee (No. 2021–451). In agreement with relevant regulatory standards, male BALB/c‐nude mice (4–5 weeks of age) were raised in a specific pathogen‐free environment. Mice purchased from SLAC Experimental Animal Corporation (Shanghai, China) were subcutaneously injected with stable SGO2‐silenced A549 cells or the controls (2.0 × 10^6^/per mouse) and randomly assigned to 4 groups (5 mice per group). Thereafter, OA at 300 mg·kg^−^^1^ or 600 mg·kg^−^^1^ was intraperitoneally delivered daily for 3 weeks as previously reported [55], and CDDP at 2.5 mg·kg^−^^1^ was intraperitoneally delivered once a week for 3 weeks [61]. Mice were euthanized for sacrifice at the experimental endpoint, and tumor xenografts, liver, and kidney were preserved for further experiments. Serum samples were collected from mice, and ALT, AST, BUN, and creatinine were measured using an automatic biochemical analyzer. The analyses were performed in a blinded manner.

### 4.20 Immunohistochemistry (IHC)

Patient consent was obtained and ethical approval were authorized by the local ethics committee (No. SHYJS‐CP‐2206002). The study included 90 paired cancer cases and 90 adjacent cancer tissues. Surgery was performed between December 2013 and July 2015, with follow‐up ranging from 5–8 years. Included were patients with surgically resected primary LUAD (pathological confirmation), paired tissues, complete data, and informed consent. Excluded were those with metastases, preoperative treatment, severe comorbidities, inadequate samples, lost follow‐up, or recent other malignancies. The Paraffin‐embedded sections of 90 LUAD patients and tumor xenografts were prepared and subjected to deparaffinization and gradient hydration. Then, antigen retrieval was conducted with citrate or Tris‐EDTA buffer followed by eradicating endogenous peroxidase with H_2_O_2_. After blocking non‐specific antigens with goat serum, the sections were treated with primary antibodies (Table S6), followed by incubation with a secondary antibody. A DAB substrate was adopted for measuring the signals. Immunohistochemically stained tissue sections were independently reviewed by two pathologists in a blinded fashion. In cases of disagreement between the two pathologists, a third pathologist was consulted to resolve the discrepancy and reach a consensus. Staining intensity was scored on a 0–3 scale: 0 (negative), 1 (weakly positive), 2 (moderately positive), and 3 (strongly positive). Staining percentage was graded on a 0–4 scale: 0 (none), 1 (1–25% of cancer cells), 2 (26–50%), 3 (51–75%), and 4 (76–100%). The final score = staining intensity × staining percentage.

### 4.21 Patient‐Derived Organoid (PDOs) Culture

Patient consent was obtained and ethical approval were authorized by the local ethics committee (No. SHYJS‐HZ‐2201). Organoids were constructed as previously reported [62]. The organoids were recovered and embedded in Matrigel (356231, Corning, USA) for culture. The organoid culture medium was replaced every 3 days. To evaluate the effect of OA on organoid growth, organoids in Matrigel were detached with TrypLE Express and resuspended in an organoid culture medium (2% Matrigel) followed by being seeded in 96‐well plates (1000 organoids/well) with low attachment round. The organoids were then treated with indicated drugs for 5 days and observed under a microscope.

### 4.22 Statistical Analysis

Statistical analysis was implemented with SPSS 22.0 and R software. From at least three independent experiments, non‐normal and normal distribution variables were expressed as the median ± range and the mean ± SD, respectively. The Wilcoxon rank‐sum test for non‐normal distribution variables and the Student's *t*‐test for normal distribution variables were utilized to elucidate statistical difference between the two groups. The statistical significance among multiple groups was estimated by the one‐way ANOVA test. The survival difference was analyzed by the log‐rank test with Kaplan‐Meier survival curves. The prognostic values of factors on LUAD patients’ survival were revealed by univariate and multivariate Cox regression models, and the hazard ratio (HR) and 95% confidence interval (CI) were calculated. *P* < 0.05 was significant.

## Author Contributions

X.L. and X.F.Z. conceived and designed the study and revised the paper. X.C., Z.D.H., C.L., M.X.Y., and B.L.Z. performed the acquisition, statistical analysis and interpretation of the data, developed the methodology and wrote the paper. X.L. and X.C. provided supervision, validation, project administration, visualization, and funding acquisition. All authors read manuscript drafts, contributed edits, and approved the final manuscript.

## Ethics Statement

Written informed consent was obtained and ethical approval were approved by the Human Research Ethics Committees of Shanghai Outdo Biotech Company (No. SHYJS‐CP‐2206002 and SHYJS‐HZ‐2201). The experimental protocols were authorized by the Institutional Animal Care and Use Committee of Shenzhen Peking University‐The Hong Kong University of Science and Technology Medical Center (No. 2021–451) and the Ethics Committee of First Affiliated Hospital of Fujian Medical University (No. [2021]479). The study was performed in accordance with the principles of the Declaration of Helsinki and complied with the International Council for Laboratory Animal Science (ICLAS) Statement on animal use in biomedical research.

## Conflicts of Interest

The authors declare no conflicts of interest.

## Supporting information




**Supporting File**: exp270188‐sup‐0001‐SuppMat.doc.

## Data Availability

The mass spectrometry data analyzed in this study have been deposited in Figshare under accession numbehttps://doi.org/10.6084/m9.figshare.32347512. Additional data supporting the findings of this study are available from the corresponding author upon reasonable request.
