# Supplementary material for: Silencing SGO2 by Oxamic Acid Dissociates Glycolysis and BRCA1‐Mediated DNA Repair to Improve the Chemosensitivity of Lung Adenocarcinoma
Source: Exploration (Beijing). 2026 May 31;6(3):20250098. doi: 10.1002/EXP.20250098 (PMC13317671; doi:10.1002/EXP.20250098)
Supplement: Supplementary file 1 — Supporting File: exp270188‐sup‐0001‐SuppMat.doc. [file EXP2-6-20250098-s001.doc]

**Supporting Information for**

**Silencing SGO2 by oxamic acid dissociates glycolysis and BRCA1-mediated DNA repair to improve the chemosensitivity of lung adenocarcinoma**

This file includes:

**Figure S1-13 and Table S1-****7.**


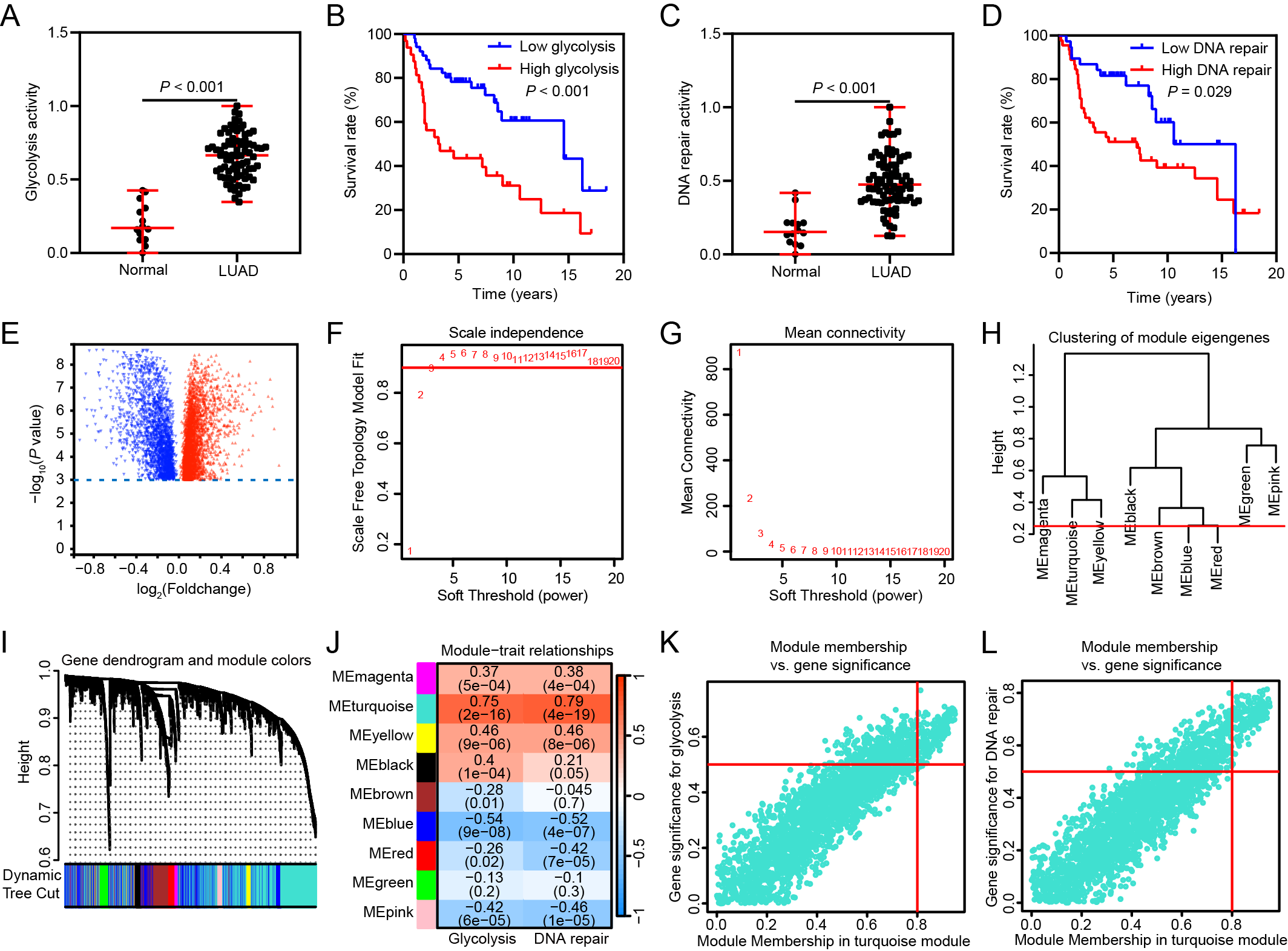


**Figure S1** Bioinformatics analyses identified glycolysis- and DNA repair-associated genes in the GSE30219 dataset. (**A**) The differential distribution of glycolysis activity in LUAD and the normal controls. (**B**) Survival analysis indicating the association between glycolysis activity and the overall survival of LUAD patients. (**C**) The differential distribution of DNA repair activity in LUAD and the normal controls. (**D**) Survival analysis showing the association between DNA repair activity and the overall survival of LUAD patients. (**E**) A volcano plot revealing the differentially expressed genes between LUAD and the normal controls. (**F, G**) The scale-free topology model fit index and soft threshold were chosen by portraying the scale independence and mean connectivity. (**H, I**) The different gene modules were displayed by the gene tree (H) and presented as the branches in the cluster dendrogram (I). (**J**) The connection between different gene modules and glycolysis or DNA repair was depicted as the correlation coefficient and (*P* value) in the heatmap. (**K, L**) The scatter plots exhibiting the modules most significantly correlated with glycolysis and repair. LUAD: lung adenocarcinoma.


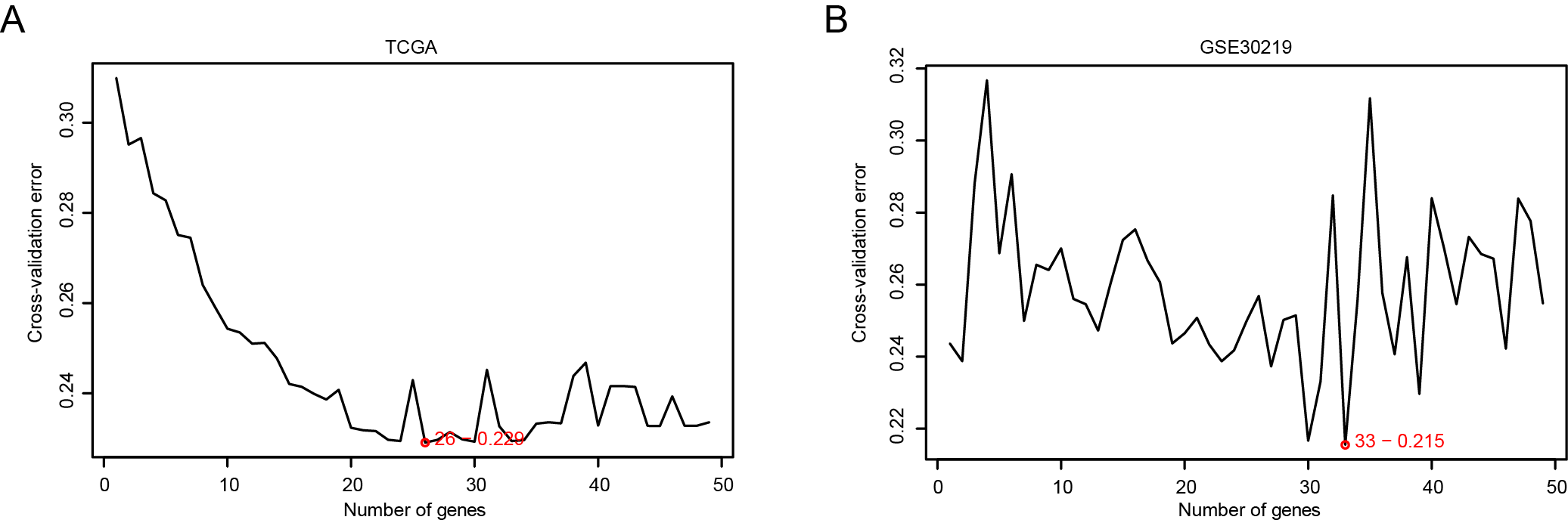


**Figure S2** The machine learning algorithm ranked the importance of overlapping genes. (**A, B**) SVM-RFE was adopted to select the most significant genes correlated with survival of LUAD patients based on the TCGA LUAD and GSE30219 datasets. LUAD: lung adenocarcinoma.

**
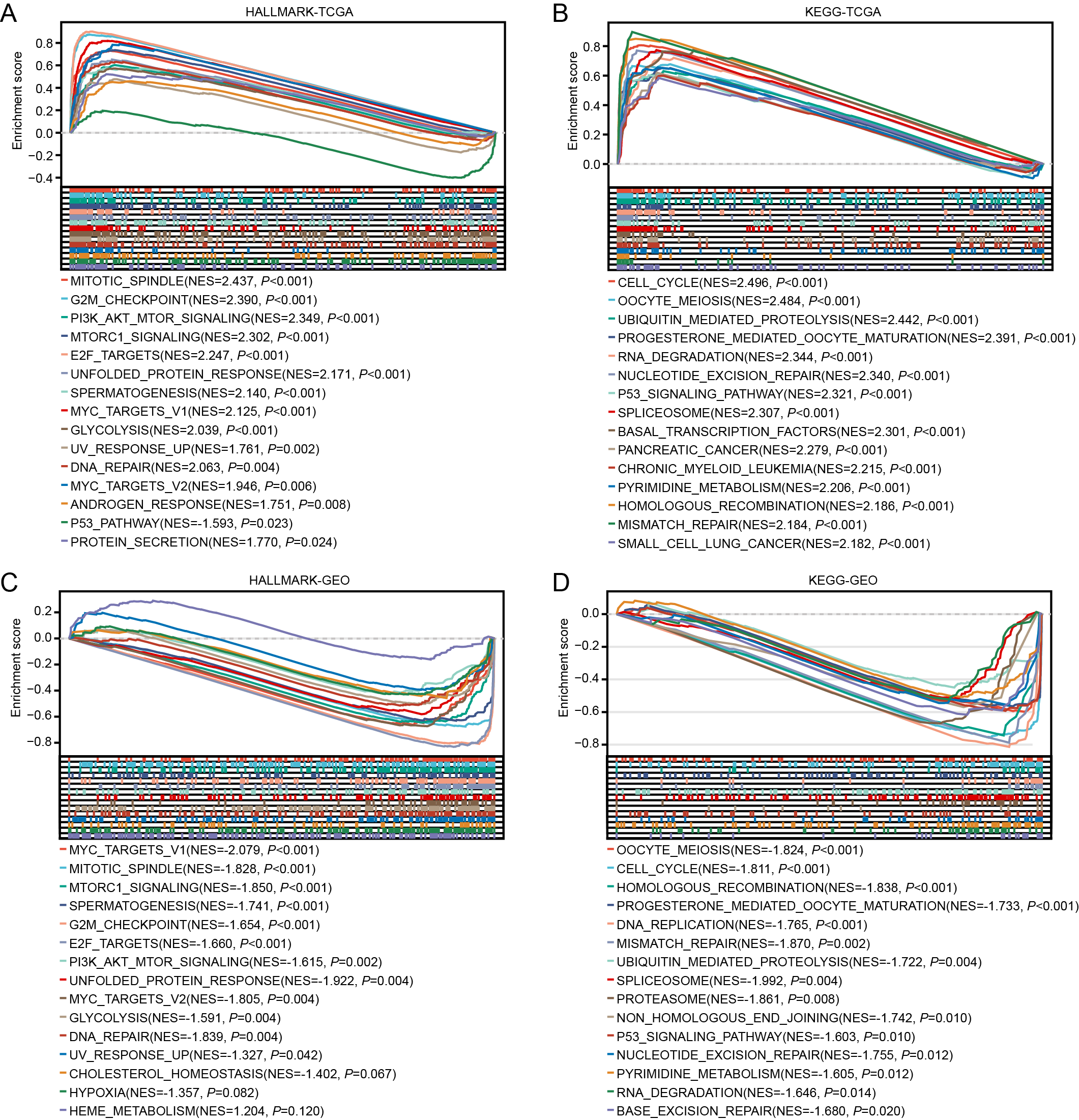
**

**Figure S3** Gene set enrichment analyses revealed the participation of SGO2 in aberrant signaling and processes as per the TCGA LUAD and GSE30219 datasets. (**A, B**) HALLMARK (A) and KEGG (B) enrichment analysis verified the participation of SGO2 in glycolysis and DNA repair signaling among the top 15 terms based on the TCGA LUAD dataset. (**C, D**) HALLMARK (C) and KEGG (D) enrichment analysis validated the participation of SGO2 in glycolysis and DNA repair signaling among the top 15 terms based on the GSE30219 dataset. LUAD: lung adenocarcinoma.

**
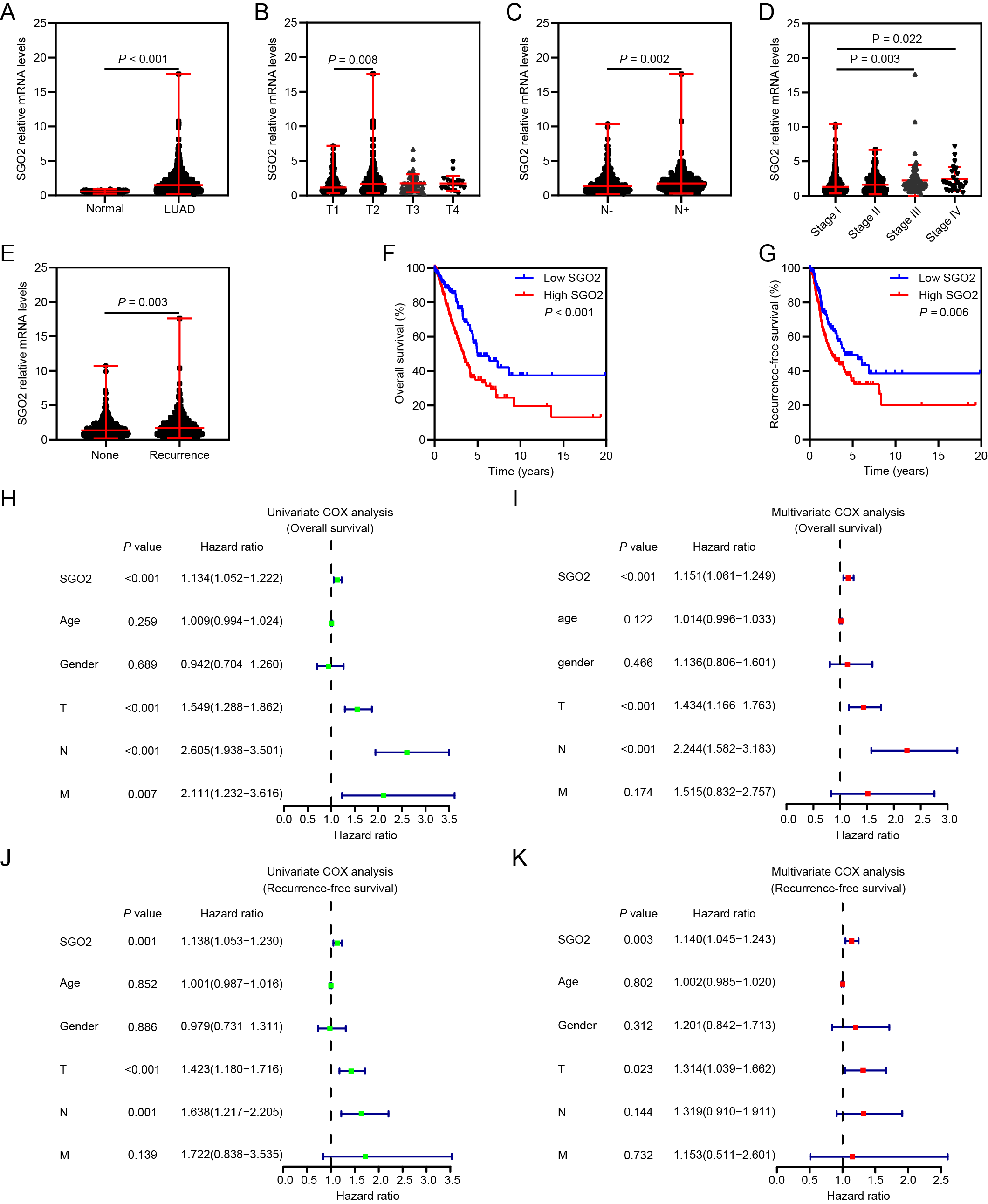
**

**Figure S4** SGO2 expression was upregulated in LUAD and predicted poor patients’ prognosis in the TCGA database. (**A**) The differential distribution of SGO2 expression in LUAD and the normal controls. (**B-E**) SGO2 expression was differentially expressed in LUAD subgroups stratified by T classification (B), N classification (C), stage (D), and recurrence status (E). (**F, G**) Survival analysis disclosing the correlation between patients’ overall survival (F), patients’ recurrence-free survival (G), and SGO2 expression in LUAD. (**H, I**) Univariate and multivariate COX analysis revealing the relationship between SGO2 expression, clinicopathological features, and the overall survival of LUAD patients. (**J, K**) Univariate and multivariate COX analysis uncovering the relationship between SGO2 expression, clinicopathological features, and the recurrence-free survival of LUAD patients. LUAD: lung adenocarcinoma.

**
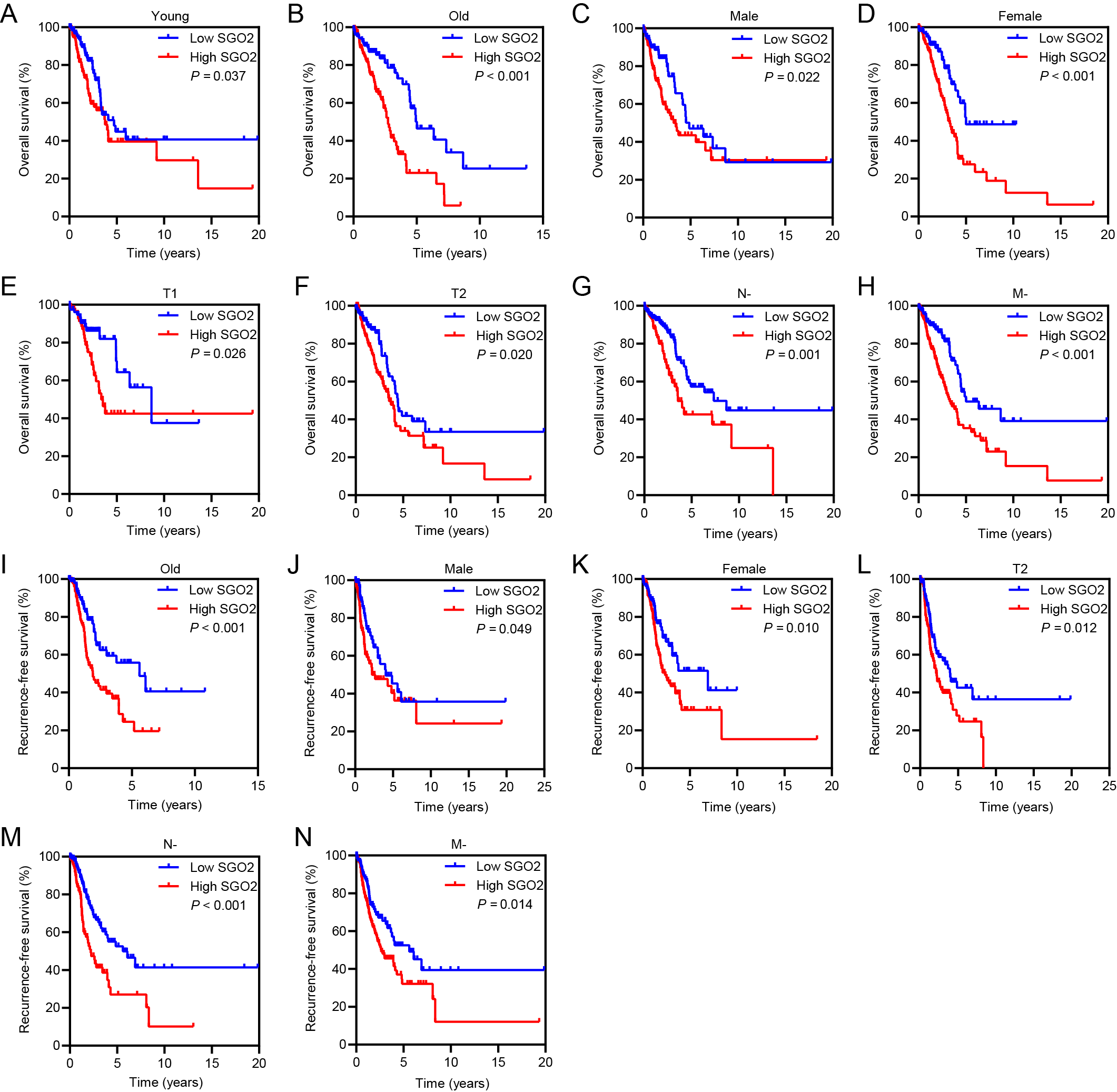
**

**Figure S5** SGO2 predicted poor prognosis of lung adenocarcinoma patients by stratified analysis as per the TCGA database. (**A-H**) Subgroup analyses disclosing the relationship between SGO2 expression and the overall survival of lung adenocarcinoma patients stratified by age (A, B), gender (C, D), T classification (E, F), N classification (G), and M classification (H). (**I-N**) Subgroup analyses revealing the association between SGO2 expression and the recurrence-free survival of lung adenocarcinoma patients stratified by age (I), gender (J, K), T classification (L), N classification (M), and M classification (N).

**
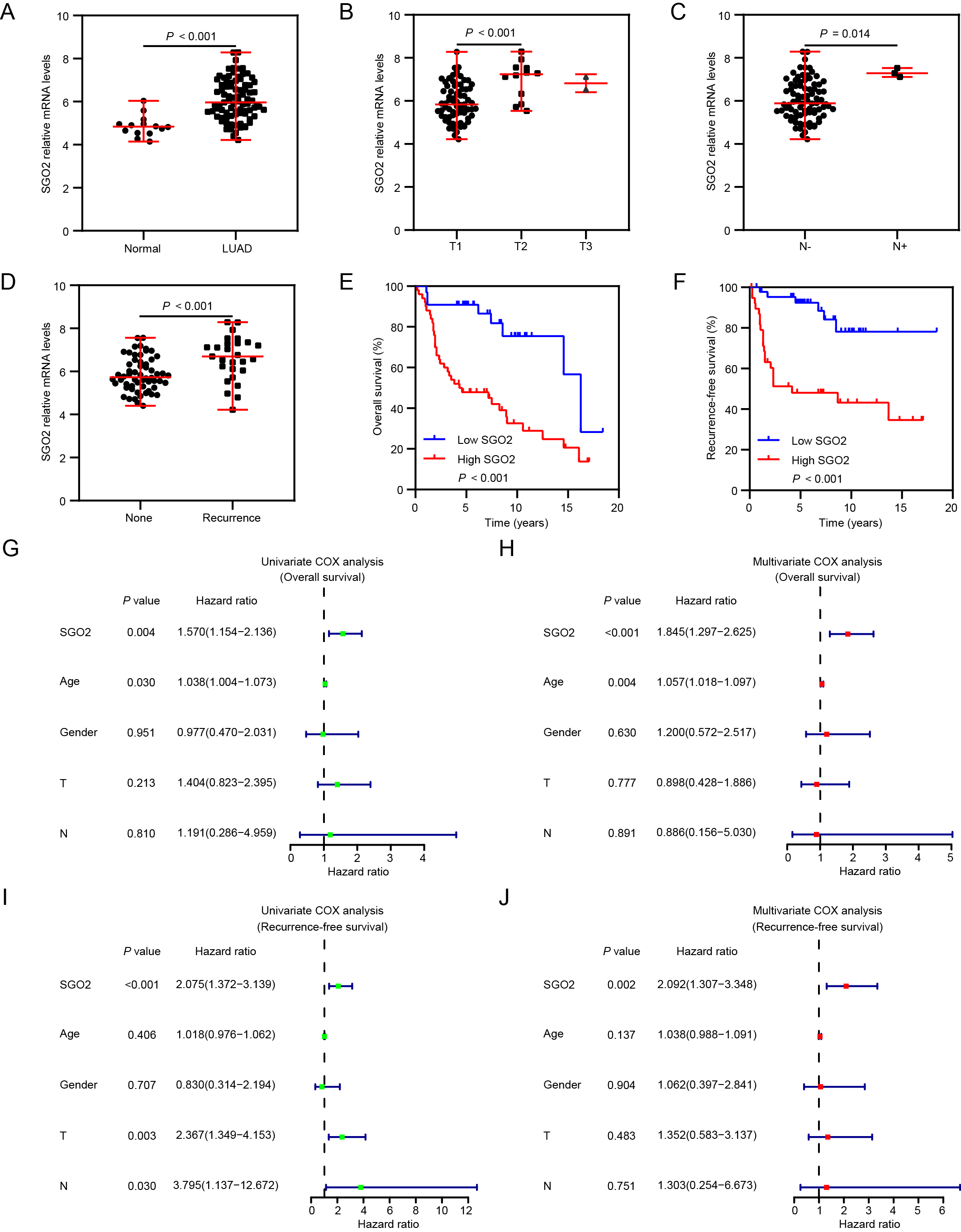
**

**Figure S6** SGO2 expression was upregulated in LUAD and conferred poor patient prognosis in the GSE30219 dataset. (**A**) The differential distribution of SGO2 expression in LUAD and the normal controls. (**B-D**) SGO2 expression was differentially expressed in LUAD subgroups stratified by T classification (B), N classification (C), and recurrence status (D). (**E, F**) Survival analysis uncovering the correlation between patients’ overall survival (E), patients’ recurrence-free survival (F), and SGO2 expression in LUAD. (**G, H**) Univariate and multivariate COX analysis revealing the relationship between SGO2 expression, clinicopathological features, and the overall survival of LUAD patients. (**I, J**) Univariate and multivariate COX analysis exhibiting the relationship between SGO2 expression, clinicopathological features, and the recurrence-free survival of LUAD patients. LUAD: lung adenocarcinoma.


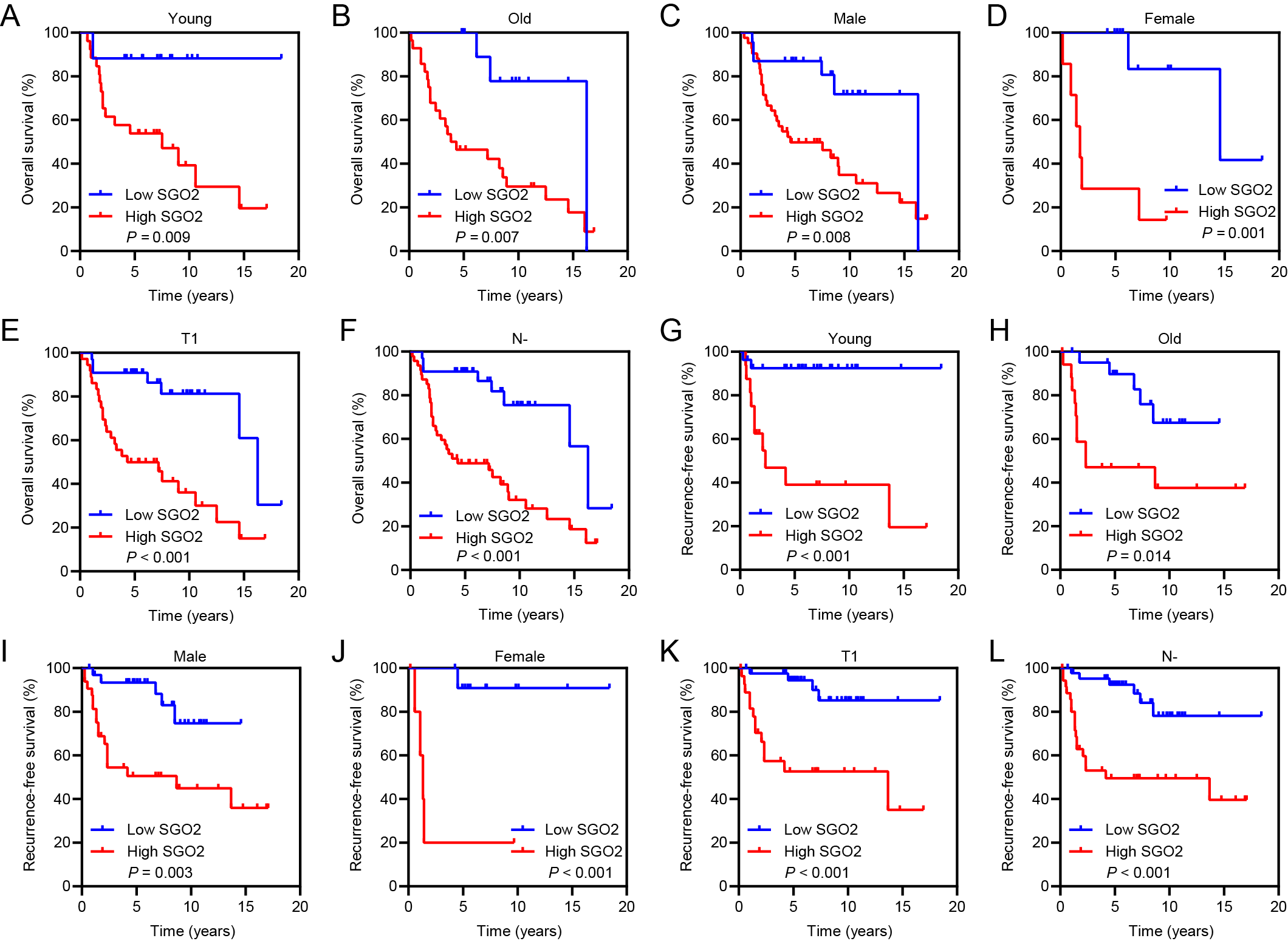


**Figure S7** SGO2 conferred poor prognosis of lung adenocarcinoma patients by stratified analysis as per the GSE30219 dataset. (**A-F**) Subgroup analyses revealing the association between SGO2 expression and the overall survival of lung adenocarcinoma patients stratified by age (A, B), gender (C, D), T classification (E), and N classification (F). (**G-L**) Subgroup analyses disclosing the relationship between SGO2 expression and the recurrence-free survival of lung adenocarcinoma patients stratified by age (G, H), gender (I, J), T classification (K), and N classification (L).


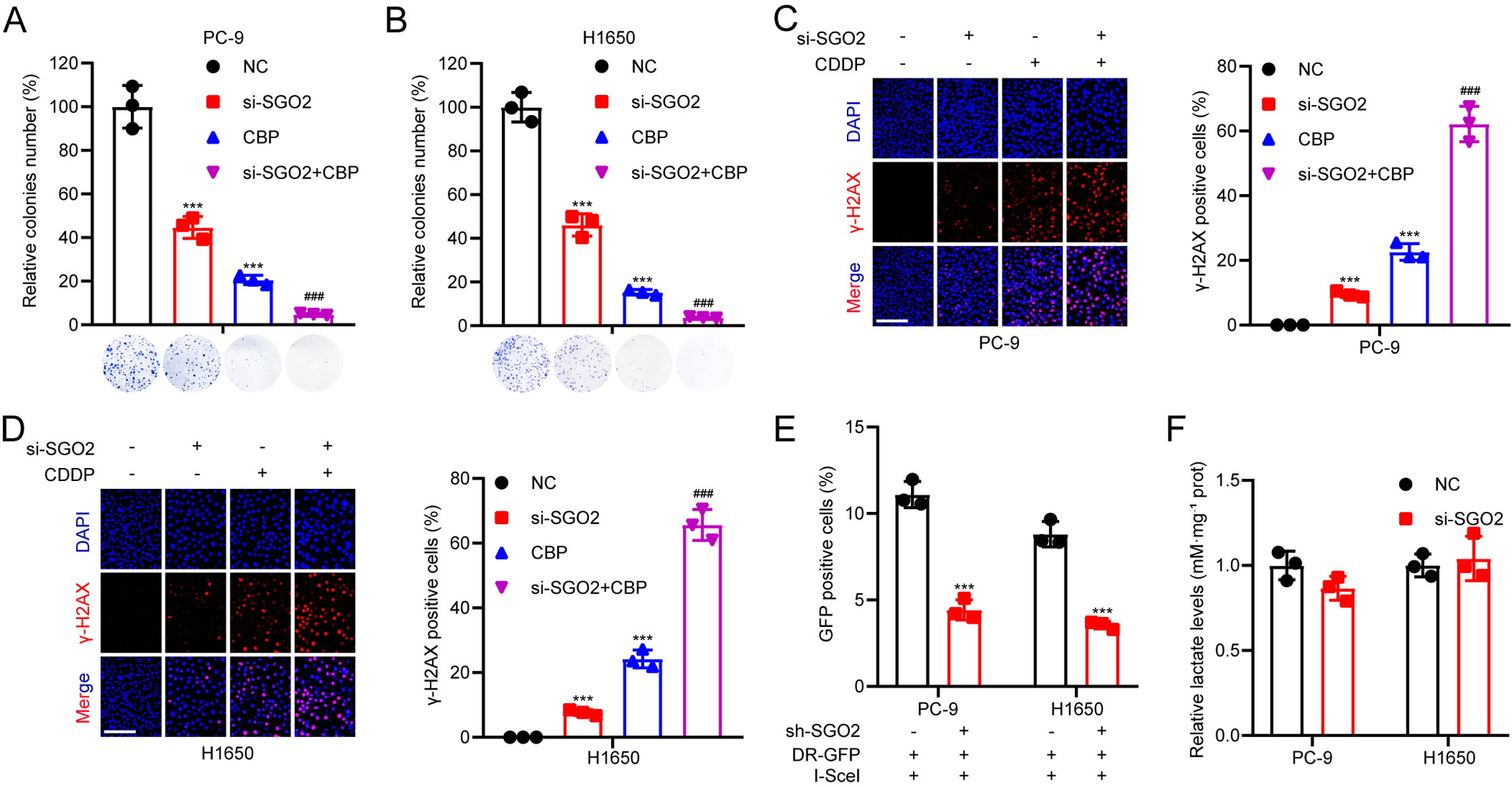


**Figure S8** SGO2 depletion improved the chemosensitivity in lung adenocarcinoma. (**A-D**) Colony formation (A, B) and immunofluorescence assays were performed to determine the chemosensitivity of SGO2-depleted PC-9 and H1650 cells, and the controls. Cells were treated with CDDP for 48 h and subjected to further analysis. Scale bars: 20 μm. *n*=3. (**E**) Homologous recombination reporter assays were applied to measure the effect of SGO2 on homologous recombination efficiency. *n*=3. (**F**) Lactate levels were detected in SGO2-depleted PC-9 and H1650 cells, and the controls. *n*=3. *** *P* < 0.001 vs. the control group. ^###^ *P* < 0.001 vs. the CDDP group. CDDP: cisplatin.

**
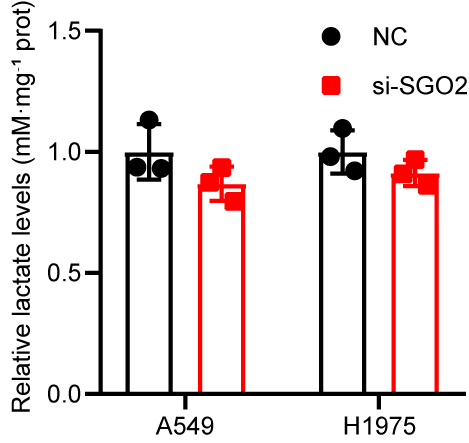
**

**Figure S9** The effect of SGO2 depletion on lactate production in lung adenocarcinoma. Lactate levels were detected in SGO2-depleted A549 and H1975 cells, and the controls. *n*=3.


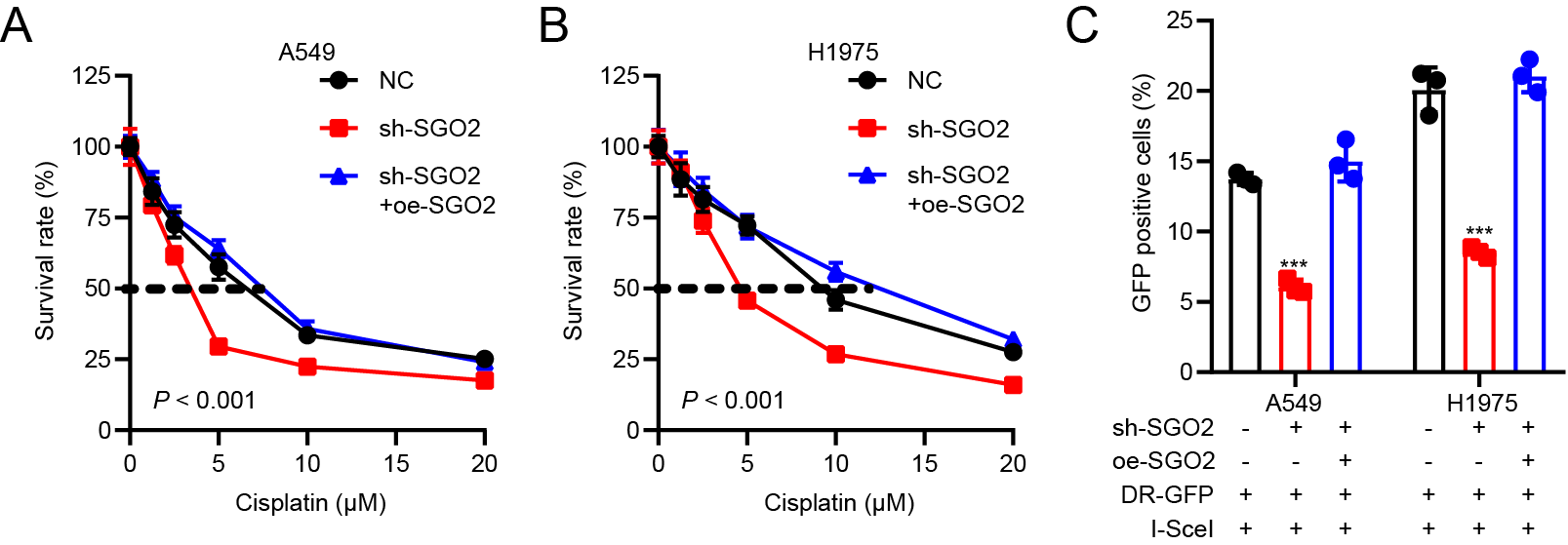


**Figure S10** SGO2 impaired the chemosensitivity in lung adenocarcinoma. (**A, B**) CCK-8 assays were performed to determine the effect of SGO2 on the chemosensitivity of A549 and H1975 cells. *n*=3. (**C**) Homologous recombination reporter assays were applied to measure the effect of SGO2 on homologous recombination efficiency. *n*=3. *** *P* < 0.001 vs. the control group.


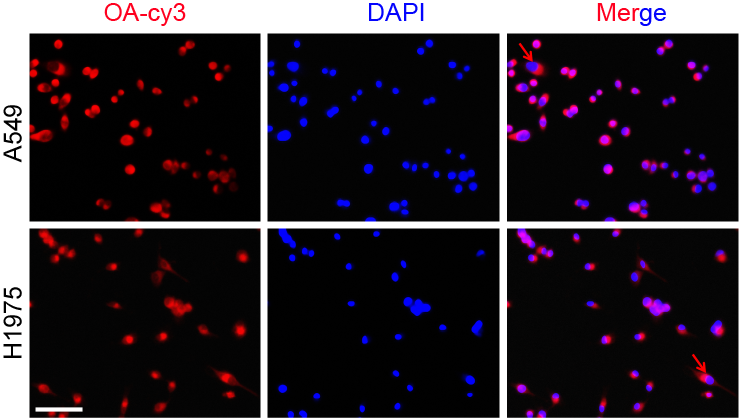


**Figure S11** OA was primarily localized in the cytoplasm after uptake. Red arrows indicated the subcellular localization of OA. Scale bars: 20 μm.


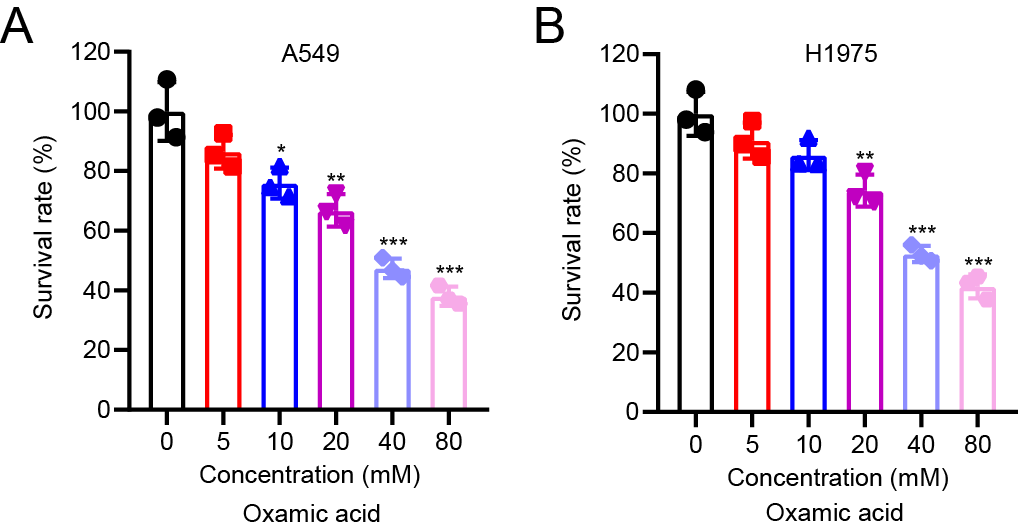


**Figure S12** Oxamic acid attenuated the viability of lung adenocarcinoma cells. (**A, B**), CCK-8 assays were conducted to determine the effect of oxamic acid on the viability of A549 and H1975 cells. Cells were treated with oxamic acid for 48 h and subjected to further analysis. *n*=3. **P* < 0.05, ** *P* < 0.01, and *** *P* < 0.001 vs. the control group.


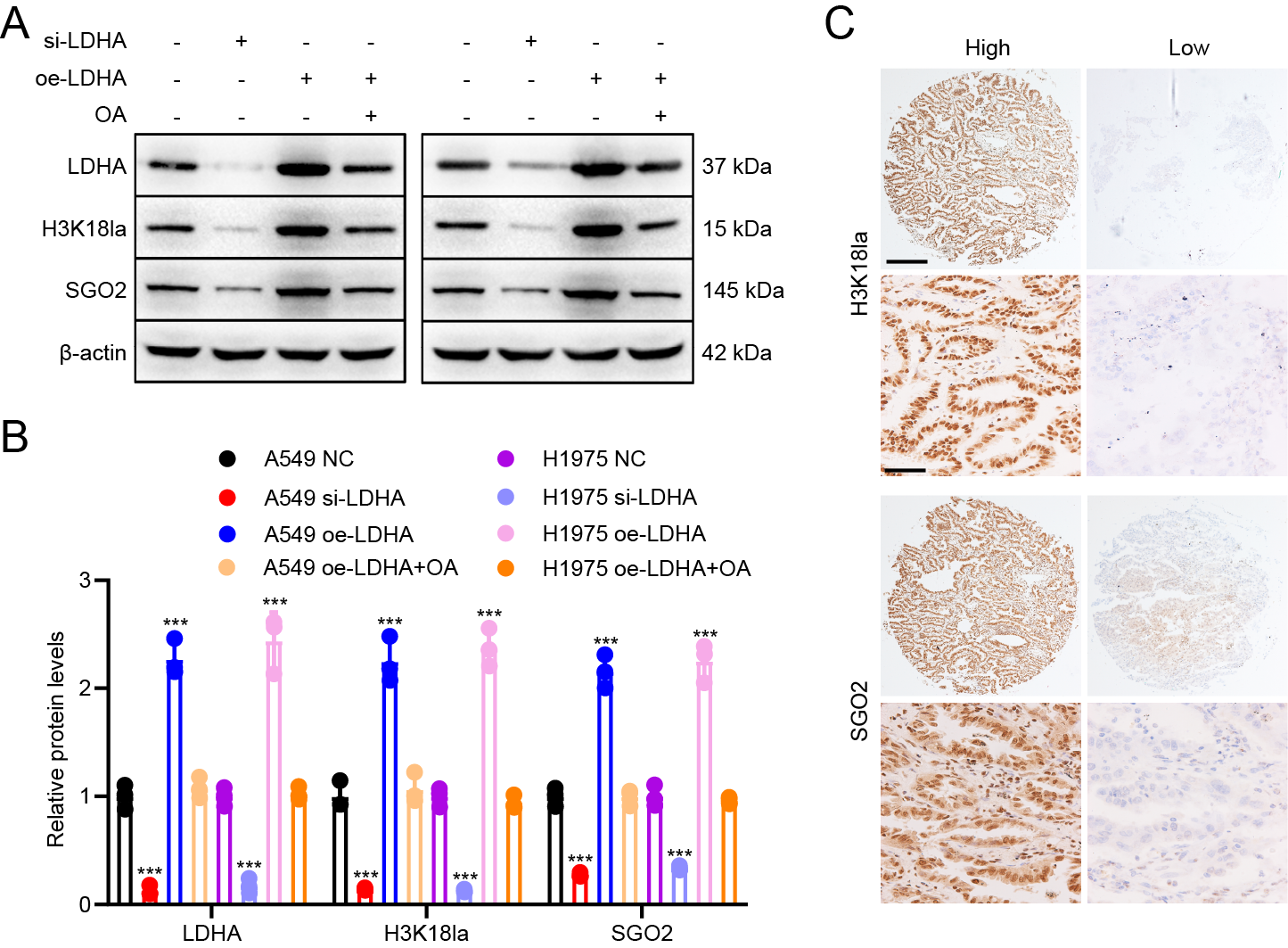


**Figure S13** Oxamic acid attenuated LDHA/H3K18la/SGO2 signaling. (**A, B**) Western blot assays were applied to disclose the impact of OA and LDHA on the expression of H3K18la and SGO2. *n*=3. (**C**) Representative images of LUAD samples indicating the correlation between H3K18la and SGO2 expression. Scale bar (upper): 100 μm; scale bar (lower): 10 μm. *** *P* < 0.001 vs. the control group.

| **Table S1** SGO2 expression in lung adenocarcinoma and adjacent normal tissues | | | | |
| --- | --- | --- | --- | --- |
| Group | Cases（n） | SGO2 expression | | *P* value |
|  |  | Low | High |  |
| Lung adenocarcinoma | 90 | 41 (45.6%) | 49 (54.4%) | ＜0.001 |
| Normal | 90 | 66 (73.3%) | 24 (26.7%) |

| **Table S2** The relationship between SGO2 expression and the clinicopathological features of lung adenocarcinoma patients | | | | |
| --- | --- | --- | --- | --- |
|
| Characteristics | n | SGO2 expression | | *P* value |
|  |  | Low | High |  |
| Age (years) |  |  |  |  |
| ≤Median | 45 | 20 (44.4%) | 25 (55.6%) | 0.832 |
| ＞Median | 45 | 21 (46.7%) | 24 (53.3%) |  |
| Gender |  |  |  |  |
| Male | 48 | 23 (47.9%) | 25 (52.1%) | 0.631 |
| Female | 42 | 18 (42.9%) | 24 (57.1%) |  |
| AJCC stage |  |  |  |  |
| Ⅰ | 44 | 22 (50.0%) | 22 (50.0%) | 0.408 |
| Ⅱ-Ⅲ | 46 | 19 (41.3%) | 27 (58.7%) |  |
| T classification |  |  |  |  |
| T1-T2 | 64 | 37 (57.8%) | 27 (42.2%) | < 0.001 |
| T3-T4 | 26 | 4 (15.4%) | 22 (84.6%) |  |
| N classification |  |  |  |  |
| N0 | 56 | 29 (51.8%) | 27 (48.2%) | 0.128 |
| N1-3 | 34 | 12 (35.3%) | 22 (64.7%) |  |
| Distant metastasis |  |  |  |  |
| No | 90 | 41 (53.7%) | 49 (46.3%) | - |
| Yes | 0 | 0 (0.0%) | 0 (0.0%) |  |

| **Table S3** The correlation between SGO2 and BRCA1 expression in lung adenocarcinoma | | | | | |
| --- | --- | --- | --- | --- | --- |
| **SGO2 expression** | **BRCA1 expression** | | **Total** | ***Kappa*** | ***P* value** |
|  | **Low** | **High** |  |  |  |
| **Low** | 33 (80.5%) | 8 (19.5%) | 41 | 0.225 | 0.018 |
| **High** | 28 (57.1%) | 21 (42.9%) | 49 |
| **Total** | 61 | 29 | 90 |  |  |

| **Table S4** The correlation between H3K18la and SGO2 expression in lung adenocarcinoma | | | | | |
| --- | --- | --- | --- | --- | --- |
| **H3K18la expression** | **SGO2 expression** | | **Total** | ***Kappa*** | ***P* value** |
|  | **Low** | **High** |  |  |  |
| **Low** | 26 (80.5%) | 19 (19.5%) | 45 | 0.244 | 0.020 |
| **High** | 15 (57.1%) | 30 (42.9%) | 45 |
| **Total** | 41 | 49 | 90 |  |  |

| **Table S5** A list of differential modification sites induced by LA based on histone modification omics | | | | |
| --- | --- | --- | --- | --- |
| Genes | Sites | Types | LA vs. NC fold change | LA vs. NC *P* value |
| H3C1 | K18 | Crotonylation | 15.8798017348203 | 5.6208E-05 |
| ZFP91 | S68 | Ubiquitin | 3.86046511627907 | 0.036481241 |
| H4C1 | R45 | Trimethylation | 0.037265625 | 2.62841E-05 |
| H2AZ1 | K7 | Acetylation (K) | 4.60510328068044 | 0.020128502 |
| H1-2 | K33 | Acetylation (K) | 0.275397796817625 | 0.046936911 |
| H4C1 | K91 | Ubiquitin | 0.123918417799753 | 0.004383456 |
| H3-3A | K27 | Acetylation (K) | 4.13065530533577 | 0.029243251 |
| H2AZ1 | K4 | Acetylation (K) | 6.61825726141079 | 0.004916078 |
| H3C1 | K36 | Methylation(KR) | 4.43445032333921 | 0.022974698 |
| EEF1A1 | K54 | Dimethylation(KR) | 0.13375 | 0.005693401 |
| H3C1 | K27 | Acetylation (K) | 3.52476669059584 | 0.04845995 |
| DEK | T12 | Phosphorylation (STY) | 42.8015564202335 | 5.40107E-08 |
| H4C1 | R67 | Methylation(KR) | 0.21047619047619 | 0.023093708 |
| HMGA1 | S43 | Phosphorylation (STY) | 0.0168421052631579 | 3.10781E-07 |
| H4C1 | K91 | Dimethylation(KR) | 0.074318744838976 | 0.000626674 |
| AHNAK | S5730 | Phosphorylation (STY) | 4.15797317436662 | 0.02860669 |
| SKIDA1 | R324 | Dimethylation(KR) | 4.92753623188406 | 0.01577334 |
| ALYREF | R196 | Methylation(KR) | 6.83229813664596 | 0.004291369 |

LA: lactate

| **Table S6** The primers used in this study | | |
| --- | --- | --- |
| Primers |  | Sequence (5’-3’ ) |
| SGO2 | Forward | CAGAAGCAAAGCACTACCACT |
|  | Reverse | CCCTGAACGCTGTCAAGTAT |
| BRCA1 | Forward | GGCTATCCTCTCAGAGTGACATTTTA |
|  | Reverse | GCTTTATCAGGTTATGTTGCATGGT |
| SGO2 promoter | Forward | GAGCCACCAGTTAGGTCAGC |
|  | Reverse | TGTTCAAGGGAGTAGAGCAGTC |
| β-actin | Forward | CTCGCTGTCCACCTTCCA |
|  | Reverse | ACCTTCACCGTTCCAGTTTT |

| **Table S7** A list of antibodies used in this study | | | | |
| --- | --- | --- | --- | --- |
| Antibodies | Cat. No | Company | Species | Application |
| SGO2 | A301-262A | Thermo | Rabbit | WB, Co-IP |
| SGO2 | 30867-1-AP | Proteintech | Rabbit | IHC, IF |
| Phospho-Histone H2A.X (Ser139) | 9718S | CST | Rabbit | IHC, IF |
| BRCA1 | 22362-1-AP | Proteintech | Rabbit | WB, Co-IP |
| BRCA1 | ab16780 | Abcam | Mouse | IHC, IF |
| FBXO44 | 10626-1-AP | Proteintech | Rabbit | WB |
| ubiquitin | 10201-2-AP | Proteintech | Rabbit | WB |
| H3K18la | PTM-1427RM | PTMBIO | Rabbit | WB, ChIP |
| H3K18la | PTM-1406RM | PTMBIO | Rabbit | IHC |
| H3K27ac | PTM-116RM | PTMBIO | Rabbit | ChIP |
| LDHA | 19987-1-AP | Proteintech | Rabbit | WB |
| β-actin | 4970T | CST | Rabbit | WB |
| Goat Anti-Rabbit IgG H&L (Alexa Fluor® 594) | ab150080 | Abcam | Goat | IF |
| Goat Anti-Rabbit IgG H&L (Alexa Fluor® 488) | ab150077 | Abcam | Goat | IF |
| Goat Anti-Mouse IgG H&L (Alexa Fluor® 594) | ab150116 | Abcam | Goat | IF |
| Flag | 66008-4-Ig | Proteintech | Mouse | WB, Co-IP |
| Flag | 20543-1-AP | Proteintech | Rabbit | WB, Co-IP |
| His | 66005-1-Ig | Proteintech | Mouse | WB, Co-IP |
| HA | 51064-2-AP | Proteintech | Rabbit | WB |
| MYC | 16286-1-AP | Proteintech | Rabbit | WB |

WB: western blot, Co-IP: Co-Immunoprecipitation, IHC: Immunohistochemistry, IF: immunofluorescence, ChIP: Chromatin immunoprecipitation.
